# Supplementary material for: Spatial-temporal characteristics and influence factors of high-quality development of animal husbandry industry in China
Source: PLoS One. 2025 Feb 4;20(2):e0313906. doi: 10.1371/journal.pone.0313906 (PMC11793817; doi:10.1371/journal.pone.0313906)
Supplement: Attachment — Submitted filename: PONE-D-24-35886.pdf [file pone.0313906.s002.pdf]

PLOS ONE

Spatial-Temporal Characteristics and Influence Factors of

high-quality development of animal husbandry industry in China

--Manuscript Draft--

Manuscript Number:	PONE-D-24-35886
Article Type:	Research Article
Full Title:	Spatial-Temporal Characteristics and Influence Factors of<br><br>high-quality development of animal husbandry industry in China
Short Title:	high-quality development of animal husbandry industry in China
Corresponding Author:	Tiantian Su<br>dongbei nongye daxue: Northeast Agricultural University<br>heilongjiang, None Selected CHINA
Keywords:	animal husbandry; high-quality development; spatial-temporal Characteristics; Spatial Association Network; influence factors; China
Abstract:	The livestock industry stands as a pillar of the agricultural sector.According to the defining characteristics of high-quality development in animal husbandry,this paper establishes a comprehensive evaluative indicator system.This system evaluates the quality of development in the industry from 2010 to 2022,including five key dimensions:output efficiency,product safety, resource conservation,environmental friendliness,and the level of scientific and technological & management.Results indicate a positive overall trend in the high-quality development of China's livestock industry throughout this period.Provinces exhibiting the highest average levels of development are,in descending order,Jiangsu,Shanghai,Anhui,Beijing,Fujian,Yunnan,Chongqing, Gansu,Guangdong,and Guangxi.Regional differences have demonstrated a slight narrowing trend; however,the effect of intra-regional differences on the overall differences has remained relatively stable.In contrast,the effect of inter-regional differences on overall differences has gradually reduced,while the effect of hypervariable density has steadily increased,becoming the most significant factor.High-quality development in China's livestock industry exhibits a strong path dependency,with advancements typically occurring incrementally between adjacent levels and an insignificant probability of leapfrogging.The level of high-quality development in a given region is significantly influenced by the development levels observed in neighbouring regions,illustrating a phenomenon akin to the adage,'.One who is near vermilion is stained red,one who is near ink is stained black' .Factors such as urbanisation rates,levels of scientific and technological innovation, transport infrastructure,levels of agricultural financial development,and population density all contribute positively to the high-quality development of China's livestock industry.Moreover,a larger share of animal husbandry in both the overall industrial sector and the agricultural industry further contributes to this high-quality development;whereas,GDP per capita and fiscal support for agriculture do not appear to have a significant effect.
Order of Authors:	Tiantian Su
	cuixia li
Additional Information:	
**Question**	**Response**
**Financial Disclosure**<br><br>Enter a financial disclosure statement that describes the sources of funding for the work included in this submission. Review	Yes

the submission guidelines for detailed requirements. View published research articles from *PLOS ONE* for specific examples.<br><br>This statement is required for submission and **will appear in the published article** if the submission is accepted. Please make sure it is accurate.<br><br>**Funded studies**<br>Enter a statement with the following details:<br>• Initials of the authors who received each award<br>• Grant numbers awarded to each author<br>• The full name of each funder<br>• URL of each funder website<br>• Did the sponsors or funders play any role in the study design, data collection and analysis, decision to publish, or preparation of the manuscript?<br><br>Did you receive funding for this work?	
Please add funding details.<br>as follow-up to "**Financial Disclosure**<br><br>Enter a financial disclosure statement that describes the sources of funding for the work included in this submission. Review the submission guidelines for detailed requirements. View published research articles from *PLOS ONE* for specific examples.<br><br>This statement is required for submission and **will appear in the published article** if the submission is accepted. Please make sure it is accurate.	This work was funded by the National Natural Science Foundation of China (The effect of infant milk powder safety trust index on product competitiveness—Index measurement, Correlation model construction and market simulation, Project number 71673042)

Powered by Editorial Manager® and ProduXion Manager® from Aries Systems Corporation

**Funded studies**<br>Enter a statement with the following details:<br>• Initials of the authors who received each award<br>• Grant numbers awarded to each author<br>• The full name of each funder<br>• URL of each funder website<br>• Did the sponsors or funders play any role in the study design, data collection and analysis, decision to publish, or preparation of the manuscript?<br><br>Did you receive funding for this work?"	
Please select the country of your main research funder (please select carefully as in some cases this is used in fee calculation).<br>as follow-up to "**Financial Disclosure**<br><br>Enter a financial disclosure statement that describes the sources of funding for the work included in this submission. Review the submission guidelines for detailed requirements. View published research articles from *PLOS ONE* for specific examples.<br><br>This statement is required for submission and **will appear in the published article** if the submission is accepted. Please make sure it is accurate.<br><br>**Funded studies**<br>Enter a statement with the following details:<br>• Initials of the authors who received each award<br>• Grant numbers awarded to each author<br>• The full name of each funder<br>• URL of each funder website<br>• Did the sponsors or funders play any role in the study design, data collection and analysis, decision to publish, or preparation of the manuscript?<br><br>Did you receive funding for this work?"	CHINA - CN
**Competing Interests**	The authors have declared that no competing interests exist

Use the instructions below to enter a competing interest statement for this submission. On behalf of all authors, disclose any competing interests that could be perceived to bias this work—acknowledging all financial support and any other relevant financial or non-financial competing interests.<br><br>This statement is required for submission and **will appear in the published article** if the submission is accepted. Please make sure it is accurate and that any funding sources listed in your Funding Information later in the submission form are also declared in your Financial Disclosure statement.<br><br>View published research articles from *PLOS ONE* for specific examples.<br><br>**NO authors have competing interests**<br><br>Enter: *The authors have declared that no competing interests exist.*<br><br>**Authors with competing interests**<br><br>Enter competing interest details beginning with this statement:<br><br>*I have read the journal's policy and the authors of this manuscript have the following competing interests: [insert competing interests here]*<br><br>* typeset	
**Ethics Statement**<br><br>Enter an ethics statement for this submission. This statement is required if the study involved:<br><br>• Human participants<br>• Human specimens or tissue<br>• Vertebrate animals or cephalopods<br>• Vertebrate embryos or tissues	N/A

• Field research<br><br>Write "N/A" if the submission does not require an ethics statement.<br><br>General guidance is provided below. Consult the submission guidelines for detailed instructions. **Make sure that all information entered here is included in the Methods section of the manuscript.**	

Powered by Editorial Manager® and ProduXion Manager® from Aries Systems Corporation

**Format for specific study types**<br><br>**Human Subject Research (involving human participants and/or tissue)**<br>• Give the name of the institutional review board or ethics committee that approved the study<br>• Include the approval number and/or a statement indicating approval of this research<br>• Indicate the form of consent obtained (written/oral) or the reason that consent was not obtained (e.g. the data were analyzed anonymously)<br><br>**Animal Research (involving vertebrate animals, embryos or tissues)**<br>• Provide the name of the Institutional Animal Care and Use Committee (IACUC) or other relevant ethics board that reviewed the study protocol, and indicate whether they approved this research or granted a formal waiver of ethical approval<br>• Include an approval number if one was obtained<br>• If the study involved *non-human primates*, add *additional details* about animal welfare and steps taken to ameliorate suffering<br>• If anesthesia, euthanasia, or any kind of animal sacrifice is part of the study, include briefly which substances and/or methods were applied<br><br>**Field Research**<br><br>Include the following details if this study involves the collection of plant, animal, or other materials from a natural setting:<br>• Field permit number<br>• Name of the institution or relevant body that granted permission	
**Data Availability**<br><br>Authors are required to make all data underlying the findings described fully available, without restriction, and from the time of publication. PLOS allows rare exceptions to address legal and ethical concerns. See the PLOS Data Policy and FAQ for detailed information.	Yes - all data are fully available without restriction

A Data Availability Statement describing where the data can be found is required at submission. Your answers to this question constitute the Data Availability Statement and **will be published in the article**, if accepted.<br><br>**Important:** Stating 'data available on request from the author' is not sufficient. If your data are only available upon request, select 'No' for the first question and explain your exceptional situation in the text box.<br><br>Do the authors confirm that all data underlying the findings described in their manuscript are fully available without restriction?	
**Describe where the data may be found in full sentences. If you are copying our sample text, replace any instances of XXX with the appropriate details.**<br><br>• If the data are **held or will be held in a public repository**, include URLs, accession numbers or DOIs. If this information will only be available after acceptance, indicate this by ticking the box below. For example: *All XXX files are available from the XXX database (accession number(s) XXX, XXX.*).<br>• If the data are all contained **within the manuscript and/or Supporting Information files**, enter the following: *All relevant data are within the manuscript and its Supporting Information files.*<br>• If neither of these applies but you are able to provide **details of access elsewhere**, with or without limitations, please do so. For example:<br><br>*Data cannot be shared publicly because of [XXX]. Data are available from the XXX Institutional Data Access / Ethics Committee (contact via XXX) for researchers who meet the criteria for access to confidential data.*<br><br>*The data underlying the results presented in the study are available from (include the name of the third party*	The data presented in this study are available on request from the corresponding author.

and contact information or URL).<br>• This text is appropriate if the data are owned by a third party and authors do not have permission to share the data.<br><br>* typeset	
Additional data availability information:	

Powered by Editorial Manager® and ProduXion Manager® from Aries Systems Corporation

Manuscript
Click here to access/download;Manuscript;high-quality of animal husbandry in China.docx

Article

Spatial-Temporal Characteristics and Influence Factors of high-quality development of animal husbandry industry in China

Tiantian Su, Cuixia Li*

College of Economics and Management, Northeast Agricultural University, Harbin 150030, China
* Correspondence:licuixia.883@163.com

Abstract: The livestock industry stands as a pillar of the agricultural sector.According to the defining characteristics of high-quality development in animal husbandry,this paper establishes a comprehensive evaluative indicator system.This system evaluates the quality of development in the industry from 2010 to 2022,including five key dimensions:output efficiency,product safety, resource conservation,environmental friendliness,and the level of scientific and technological & management.Results indicate a positive overall trend in the high-quality development of China's livestock industry throughout this period.Provinces exhibiting the highest average levels of development are,in descending order,Jiangsu,Shanghai,Anhui,Beijing,Fujian,Yunnan,Chongqing, Gansu,Guangdong,and Guangxi.Regional differences have demonstrated a slight narrowing trend; however,the effect of intra-regional differences on the overall differences has remained relatively stable.In contrast,the effect of inter-regional differences on overall differences has gradually reduced,while the effect of hypervariable density has steadily increased,becoming the most significant factor.High-quality development in China's livestock industry exhibits a strong path dependency,with advancements typically occurring incrementally between adjacent levels and an insignificant probability of leapfrogging.The level of high-quality development in a given region is significantly influenced by the development levels observed in neighbouring regions,illustrating a phenomenon akin to the adage,'.One who is near vermilion is stained red,one who is near ink is stained black' .Factors such as urbanisation rates,levels of scientific and technological innovation, transport infrastructure,levels of agricultural financial development,and population density all contribute positively to the high-quality development of China's livestock industry.Moreover,a larger share of animal husbandry in both the overall industrial sector and the agricultural industry further contributes to this high-quality development;whereas,GDP per capita and fiscal support for agriculture do not appear to have a significant effect.

Keywords:animal husbandry;high-quality development;spatial-temporal Characteristics;Spatial Association Network;influence factors;China

1. Introduction

Driven by increasing demand for meat,eggs,milk,and other livestock products, China's livestock industry has become a cornerstone of its agricultural sector, representing 34% of agricultural output[1].This sector plays a critical role in stimulating rural economic growth,enhancing farmers' incomes,ensuring national food security, and achieving agricultural and rural modernisation.However,under the pressure of increasing resource constraints,China's animal husbandry industry continues to suffer from low production efficiency,a large feed-grain deficit,severe environmental pollution, limited technological advancement,and the unsustainable utilisation of grassland resources[2-4].Moreover,the upgrading of China's consumption patterns and rising living standards have fuelled a bur-

geoning demand for green,high-quality,and safe livestock products,placing greater demands and challenges on the industry.Therefore, conventional livestock production methods have proven inadequate in meeting the population's health,safety,and environmental needs,making the transition from rudimentary practices to an intensive,high-quality development model a necessary[5]. High-quality development in animal husbandry represents a crucial strategic programme introduced by China in its new phase of development.This programme specifically addresses the problem of development impetus by innovation,the problem of unbalanced development by coordination,the problem of harmony and coexistence between human beings and nature by greenery,the problem of internal and external linkage of the country by opening up,and to solve the problem of social harmony by sharing[6].In 2020,the General Office of the State Council released the"Opinions on Promoting the High-Quality Development of the Animal Husbandry Industry," advocating for the advancement of the sector towards a new paradigm characterised by high-efficiency outputs,product safety,resource conservation,environmental friendliness, and effective governance.[7].In addition,in 2023,the State also amended the Livestock Law of the People's Republic of China in terms of doing a good job of harmless treatment of livestock and poultry manure,promoting the balance of grass and livestock,and strengthening the green development of the livestock industry,aiming to regulate the production and operation of the livestock industry and promote the high-quality development of the livestock industry.So,what is the current state of high-quality development in China's animal husbandry industry following collaborative efforts by the government,societal organisations,farmers,and other stakeholders?What are the characteristics of temporal evolution?What kind of spatial correlation characteristics exist in the high-quality development of China's livestock industry?What are the characteristics of regional differences?What factors influence the level of high-quality development in China's livestock industry?By quantitatively analysing these issues based on a scientifically established evaluation index system for high- quality animal husbandry in China,this paper aims to offer valuable theoretical insights to promote high-quality development of animal husbandry industry in China.

At present,most scholars have focused mainly on studies related to the level of high-quality development in agriculture.;whereas,analyses concerning high-quality development in the livestock sector remain limited.On the qualitative side,scholars mainly summarise the achievements and problems faced by China's animal husbandry industry,the connotation of high-quality development of animal husbandry and the path to achieve it.Since the reform and opening up,China's animal husbandry industry has made great achievements.Productivity has risen dramatically,with per capita meat,egg and milk consumption already at 140.2%,213.2% and 28.0% of the world average, respectively.The structure of the industry is increasingly optimised,with the proportion of beef,mutton and poultry meat in meat consumption gradually increasing.The level of scale is steadily increasing,with the scale of livestock and poultry farming reaching 67.5% in 2020.The support and guarantee system is also improving day by day. However,the development of animal husbandry in China is still facing many challenges, such as tighter resource constraints,stricter environmental protection constraints,more severe epidemic prevention and control,high degree of external dependence on core technologies and weak international competitiveness.High-quality development of the livestock industry is an important strategic initiative to meet the challenges,and its connotation has been interpreted by different scholars from different perspectives. Generally speaking,it is to achieve high quality in the production process and results. The high quality of the production process is to take green development as the background colour,science and technology as the driving force,a safe and sufficient forage system and a high degree of attention to animal welfare as the breeding environment,and large-scale,standardised and industrialised as the mode of production, and then to build up an intensive mode of production that is high production efficiency, high economic efficiency,high level of resource conservation and

environmental friendliness.The high quality of the production results is to meet the comprehensive, diversified and personalised demand of the residents for livestock products,and to provide the residents with safe and high-quality livestock products[8-12].

In the quantitative aspect,scholars mainly focus on the evaluation of the development of livestock modernisation and the evolution of spatio-temporal dynamics. In the evaluation of indicators,different dimensions of indicator construction lead to different measurement results.Domestic scholars mainly focus on five dimensions of material and equipment level,scientific and technological progress level,operation and management level,safe and sustainable level,and industrial output level [13];five dimensions of green development,industrial output,scientific and technological progress, material and equipment,and operation and management level[14];four dimensions based on the industrial chain perspective from the upstream,midstream, downstream, and resourcefulness utilisation[15];and four dimensions of resource conservation, environmental friendliness,product safety,and output efficiency[16] to construct the index system of animal husbandry modernisation or green development level.In terms of spatial and temporal evolution characteristics,the Gini coefficient and kernel density maps are mainly used to reveal their dynamic evolution characteristics.In terms of high-quality development of the livestock industry,only Xiong Xuezhen (2022) [17]measured the level of high-quality development of the livestock industry from three dimensions:green cycle development,supply quality and efficiency,and optimisation of business management.Few scholars have explored the influencing factors on the high-quality development of animal husbandry,and most scholars have explored the influence of urbanisation rate,transport level,scientific and technological innovation level,digital economy development level,financial support for agriculture level, environmental regulation,industrial structure,rural finance and so on[18-22]on the high- quality development of agriculture,which provides theoretical references to the research of this paper.

In summary,drawing upon the"Opinions on Promoting High-Quality Development of Animal Husbandry,"this paper proposes a novel method for evaluating the high- quality development of this industry in China,comprising five key dimensions:high output efficiency,product safety,resource conservation,environmental sustainability,and level of scientific and technological &management.It is important to note that due to the challenges in obtaining data related to the regulation of effective indicators,the fifth dimension of this paper focuses specifically on measuring the level of existing science, technology and management practices in China's livestock industry.

2. Materials and Methods

2.1. Research Methods

2.1.1.Entropy weight comprehensive evaluation method

The entropy weight method offers a comprehensive means of evaluating situations characterised by multiple indicators and characteristics.The method carries out comprehensive evaluation by considering the correlation and importance between indicators and determining the weight of each indicator.The entropy weight method can be broken down into the following steps:

(1) Indicator Identification and Data Acquisition:Begin by identifying the specific indicators requiring evaluation and collect the relevant data.

(2) Data Standardisation:Standardise the data for each indicator to ensure comparability across the dataset.

(3) Entropy Value Calculation:Calculate the entropy value for each indicator.This value reflects the information content and uncertainty associated with each indicator.

(4) Weight Calculation:Derive the weight of each indicator from its corresponding entropy value. The entropy weight method hypothesizes that indicators with higher entropy values should be assigned greater weights, and vice versa.

(5) Comprehensive Evaluation:To arrive at a weighted score for each indicator, multiply its weight by its standardised value.Sum these weighted scores across all indicators to produce the final comprehensive evaluation result.

The entropy weight method is highly effective because it considers the weight and information content of each indicator,circumventing the pitfalls of subjective weight assignment.Therefore,it enjoys widespread application in multi-indicator decision- making and evaluation processes [23-24].

2.1.2.Dagum Gini coefficient and decomposition method

The Dagum Gini coefficient represents an optimization of the traditional Gini coefficient.Its key advantage is reflected in its decomposability into intra-group coefficient,inter-group coefficient and hypervariable density coefficient.By addressing the issue of overlapping data,which often limits other regional difference measures,the Dagum Gini coefficient offers superior identification of the root causes driving regional differences. The formula is:

$$G=\frac{\sum_{j=1}^{k}\sum_{h=1}^{k}\sum_{i=1}^{n_j}\sum_{r=1}^{n_k}\left| y_{ji}-y_{hr}\right|}{2n^2\overline{y}}$$

Where k is the number of regional divisions, n represents the number of provinces in each region, n_j, n_h represent the number of provinces in the region j, h. y_{ji}, y_{hr} represent the high quality level of animal husbandry in provinces i, r in regions j,h. $\overline{y}$ denotes the average level of high-quality development of China's livestock industry.The Dagum Gini coefficient can be decomposed into three components:the contribution of intra-regional differences (G_W),the contribution of inter-regional differences (G_{nb}),and the contribution of hypervariable density (G_t),and satisfy G = $G_W+G_{nb}+G_t$ [25-26].

2.1.3.Kernel density estimation method

Kernel density estimation is a non-parametric statistical method used to estimate the probability density function of a random variable.This method estimates the overall probability density function by placing a kernel function (usually a Gaussian kernel function or a rectangular kernel function) around each data point,and then weighting these kernel functions to estimate the overall probability density function,which is effective in observing the dynamic evolution of the sample data[27].Assume that the density function of the random variable x is:

$$f(x)=\frac{1}{Nh}\sum_{I=1}^{N}K\left(\frac{x_i-x}{h}\right)$$

Where,N is the number of observations, x_i denotes the observation value,x is the mean value,K(·) represents the Kernel density,and the bandwidth is denoted by h.Consistent with most studies,in this paper,Gaussian kernel function is chosen to estimate the distributional dynamics of high quality development of China's animal husbandry industry, see equation (2)

$$k(x)=\frac{1}{\sqrt{2\pi}}\exp\left(-\frac{x^2}{2}\right)$$

In essence,kernel density estimation offers a flexible and robust statistical method for understanding the distributional characteristics and probability density functions in data.

2.1.4.Markov chain analysis

Markov chain analysis,a mathematical tool, is utilised to study stochastic processes. Specifically, it focuses on processes where the probability of transitioning between states depends solely on the present state,independent of past states – a property termed the Markov property. In this analysis,a system can exist in a range of possible states,with the transition probabilities between these states determined by a predefined probability distribution.By modelling and analysing these state transfer probabilities,we can gain a

deeper understanding of the dynamic evolution laws and characteristics of China's livestock industry's development towards high quality.

$$p\{X(t)=j \mid X(t-1)=i, X(t-2)=i_{t-2},\ldots,X(0)=i_0\}=P\{X(t)=j \mid X(t-1)=i\}$$

From equation (4), the state of random variable X at a future time period t is solely influenced by its state one period prior to t, i.e.,X has the property of first-order Markov. In addition,the transfer probability p_{ij} of random variable X from state i to state j can be obtained by n_{ij}/n_i. n_{ij} denotes the total number of times state i transitions into state j,and n_i is the number of times state i occurs.. Therefore, by analysing the state dimension matrix P, constructed from these transition probabilities, we can infer the dynamic evolution trends in the distribution of high-quality development in China's animal husbandry industry [28-29].

2.2.Construction of the indicator system

With Chinese socialism entering a new era,the nation’s economic development has transitioned from an emphasis on“quantity”to one on“quality”.The proposition of high-quality development in animal husbandry not only speaks to the burgeoning desire of the populace for an improved standard of living,but also represents an essential strategy for addressing the pressures of resource scarcity and environmental degradation.This paper will concentrate on the following five aspects to construct evaluative indicators:

First,the level of output efficiency.Considering existing resource constraints, improvements to production efficiency are essential if we [illegible] to meet the ever-growing demand for products.For the livestock industry,achieving greater output efficiency signifies producing a higher output utilizing the same or even fewer resource inputs. This naturally results in the production of greater quantities of meat,eggs,milk and other valuable livestock products.From an economic perspective,this improvement in output efficiency translates to lower feed costs,labour costs,and other expenditures related to farming.It also leads to the optimisation of resource allocation and the maximisation of economic benefits.This paper selects five key indicators to measure the output efficiency of China's livestock industry:livestock slaughter rate,cost profitability,production efficiency,the level of meat consumption,and the level of milk consumption.

Second,the level of product safety.This signifies that livestock products must be free from harmful substances,remain uncontaminated and unspoiled,adhere to relevant health standards and legal and regulatory requirements,and finally,protect consumer health and safety.More specifically,additives,antibiotic residues,heavy metals,and other harmful substances present in feed will affect the safety of livestock products.The unreasonable utilisation of veterinary drugs,antibiotics,and other medications may lead to excessive residues in livestock products,which can,accordingly,harm human health. In addition,the hygienic conditions of the breeding environment are directly correlated with the quality and safety of livestock products.Epidemic outbreaks can result in health issues for livestock and poultry,thereby affecting the quality and safety of the products derived from them.Considering the available data,this article utilises the following factors to measure the quality and safety level of the animal husbandry industry [30]:the cost rate of epidemic prevention;the level of green food production;the qualification rate of livestock and poultry product quality and safety monitoring;the qualification rate of feed product quality and safety monitoring;and the proportion of senior and mid-level technicians among the total number of staff at township animal husbandry and veterinary stations.

Third,the level of resource conservation.Livestock farming necessitates large inputs of resource allocation,including water,land,feed,and energy.Therefore,prioritising resource conservation can reduce consumption without compromising output. Regarding feed,optimisation of formulations and processing methods can enhance utilisation.For water resources,implementing recycling systems such as rainwater harvesting and efficient irrigation is crucial to minimise wastage.Land resources are best managed through

intensive farming practices,such as ecological animal husbandry and recycling agriculture,to maximise the utility of finite land.In addition,integrating high- efficiency energy equipment and technologies,including energy-saving light sources and solar energy utilisation,effectively reduces energy consumption throughout the livestock production process.This paper employs five indicators to measure the effectiveness of resource conservation in animal husbandry,contingent upon data availability:the level of intermediate consumption resource use,the level of feed resource use,the efficiency of feed grain production,the level of labour resource use,and the level of land resource use.

Fourth,the level of environmental friendliness.according to the Second National Pollution Source Census Bulletin,the livestock industry discharged 10,005,300 tonnes of chemical oxygen demand (COD),110,900 tonnes of ammoniacal nitrogen,596,300 tonnes of total nitrogen,and 119,700 tonnes of total phosphorus in 2017.These figures represent 93.76%,51.30%,42.14%,and 56.46% of the total water pollution from agricultural sources, respectively.In addition,projections indicate that livestock carbon emissions constituted 33.46% of total agricultural carbon emissions in 2019.Therefore,the livestock industry remains a significant contributor to agricultural surface pollution in China.Expediting the green transformation of this industry is essential for its high-quality advancement. For instance,resource recovery from manure is achievable through biogasification and other treatment methods,reducing pollution risks to soil and water bodies. Promoting ecological farming models such as grassland grazing and circular agriculture is also vital to protect natural ecosystems and minimise ecological damage.Moreover,the nation must prioritise the adoption of environmental protection technologies and equipment. This includes energy-saving emission reduction equipment,sewage treatment facilities, and the utilisation of clean energy to reduce the environmental effects of livestock production and achieve greener practices.This paper employs five indicators to assess the environmentally friendly development level of China's livestock industry:the comprehensive utilisation rate of livestock and poultry manure,the level of household biogas utilisation,total carbon emissions,the carrying capacity of land for livestock and poultry farming, and green total factor productivity [31-32].

Fifth,the level of scientific and technological management.Advances in information technology have positioned scientific and technological innovation as a prerequisite for enhancing productivity and cultivating high-quality development in the livestock industry.For instance,integrating technologies such as the Internet of Things,big data analytics,and artificial intelligence allows for intelligent monitoring and management of livestock production processes.accuracy feeding technology enables the accurate deployment of feed formulas,optimising feed utilisation.In addition,environmental monitoring technology facilitates real-time tracking of temperature and humidity levels in livestock barns,permitting timely adjustments to maintain optimal conditions. However,the traditional family-based smallholder production model is incompatible with the high-quality development in China's livestock industry.Eliminating outdated production capacity and expediting the transition towards large-scale livestock and poultry farming are essential steps.Such a shift facilitates the adoption of advanced production technologies and management models,thereby enhancing production efficiency.Moreover,it cultivates economies of scale,reduces production costs,and thus enhances economic efficiency.It is important to recognise that effective management is contingent upon human capital.A highly skilled workforce with a strong environmental awareness and learning capacity is more adept at managing livestock farms.This study employs indicators such as the intensity of mechanical equipment,the scale of livestock and poultry farming,labour force quality,the proportion of senior workers in animal husbandry stations,and the proportion of breeding livestock and poultry stock to comprehensively assess the level of scientific and technological &management in livestock and poultry farming.

The data in this paper are mainly from official statistical yearbooks,including China Statistical Yearbook,China Rural Statistical Yearbook,China Animal Husbandry and Vet-

erinary Yearbook,China Environmental Statistical Yearbook,as well as statistical yearbooks and animal husbandry and veterinary yearbooks of each province.According to the division criteria of the National Bureau of Statistics,the eastern region includes Beijing,Tianjin,Hebei,Liaoning,Shanghai,Jiangsu,Zhejiang,Fujian,Shandong,Guangdong, and Hainan;the central region includes Shanxi,Jilin,Heilongjiang,Anhui,Jiangxi,Henan, Hubei,and Hunan;and the western region includes Inner Mongolia,Guangxi,Chongqing, Sichuan,Guizhou,Yunnan,Shaanxi,Gansu,Qinghai,Ningxia,and Xinjiang.

Table 1. Indicator system for evaluating the level of high-quality development of the livestock industry

Primary indicators	Secondary indicators	Interpretation of indicators	Indicator properties
Level of output efficiency	Livestock slaughter rate	Number of animals slaughtered in the period/Stock at beginning of period	+
	cost margin	Net profit/total cost	+
	Level of meat consumption	Per capita meat consumption	+
	Level of milk consumption	Per capita milk consumption	+
	production efficiency	The input indicators are the number of labourers in the livestock industry, intermediate consumption in the livestock industry, and investment in fixed assets in the livestock industry, and the output indicator is the total output value of the livestock industry, which is calculated by applying the super-efficiency SBM model, and the details of the calculation methodology can be found in the study by Chun Yang (2019)[33]	+
Level of Product safety	the cost rate of epidemic prevention	Epidemic defence cost/production costs	+
	Level of green food production	Number of green food certifications	+
	the qualified rate of livestock and poultry product quality and safety monitoring	Quantity of qualified products/total quantity of products	+
	The qualified rate of feed product quality and safety monitoring	Quantity of qualified products/total quantity of products	+
	Share of senior and middle-level technicians in the number of staff on board at township animal husbandry and veterinary stations	Total number of senior and middle-level technicians/Total number of staff on board at livestock and veterinary stations	+
Level of resource conservation	Level of intermediate consumption resource use	Gross value of livestock production/Pastoral intermediate consumption	+
	Level of utilisation of feed resources	Livestock output/feed industry output	+
	Efficiency of feed grain production	The input indicators include the amount of fertiliser applied for grain production,the total power of machinery for grain production,and the area sown for grain,while the output indicator is the total grain output,which is calculated by using the super-efficient SBM model,and the details of the calculation method can be found in the study by Chuanming Liu (2023)[34]	+

	Level of utilisation of labour resources	Gross value of livestock production/number of labourers employed in the livestock sector	+
	Level of land resource use	Gross value of livestock production/area of pasture land	+
	Level of resource use of livestock and poultry manure	Comprehensive livestock and poultry manure utilisation rate	+
	Level of household biogas utilisation	Rural household biogas production/number of rural population	+
	Carbon emission levels	Carbon emissions from faecal and gastrointestinal fermentation (calculated based on IPCC coefficients), see Jinxin Zhang (2020) for details of the calculation process.[35]	−
Level of environmental friendliness	Livestock manure land carrying capacity	Calculated according to the calculation method in the Technical Guidelines for Measuring the Land Carrying Capacity of Livestock and Poultry Manure	+
	Green total factor productivity	The input indicators are the number of labourers in the livestock industry, intermediate consumption in the livestock industry, and fixed asset investment in the livestock industry, the desired output indicator is the total output value of the livestock industry, and the non-desired output indicator is the carbon emission, which is calculated by using the super-efficiency SBM model, and the method of calculation is detailed in the study by Biaowen Xu (2019).[36]	+
	Mechanical equipment strength	Number of units of livestock (breeding) machinery/total livestock production	+
	Degree of livestock and poultry farming scale	Number of large-scale farms (households)/total number of farms (households)	+
Level of science and technology & management	Quality of labour force	Average level of education of agricultural labourers	
	Percentage of senior staff at livestock stations	Number of senior employees in livestock stations / Total number of employees in animal husbandry stations	+
	Percentage of stock of breeding animals	Stock of breeding animals/Total stock of livestock and poultry	+

Note:In this paper, the scale level is determined according to the criteria of annual pig slaughtering⩾500,dairy cattle stocking⩾100,beef cattle stocking⩾100,sheep stocking⩾100,egg-laying chicken stocking ⩾2000,broiler stocking ⩾5000.

3. Results

3.1. Descriptive statistics on the high-quality development of China's animal husbandry industry

The overall standard of the high-quality development of animal husbandry in China's provinces remained between 0.09 and 0.35 throughout the period from 2010 to 2022.Across the 30 provinces,the average level of high-quality development in the livestock industry has exhibited an upward trend,rising from 0.13 in 2010 to 0.17 in 2022. Analysing specific dimensions,the level of scientific and technological &management in China's livestock and poultry farming demonstrates a clear upward trend,achieving the highest level across all five dimensions.The level of resource conservation has remained relatively consistent,maintaining a relatively high level across the five dimensions. Environmental performance demonstrates a clear pattern of improvement,reflecting the in-

creasing effectiveness of environmental management strategies in the livestock and poultry industry and the positive effect of sustainable livestock farming practices.The level of output efficiency has remained stable but at a lower level,suggesting that there is significant potential for improvement in the productivity of China's livestock and poultry farming.The safety of livestock products,while currently at a relatively low level, exhibits a clear upward trend.This suggests that the increasingly rigorous oversight of livestock production in China,coupled with stringent measures against the prohibited drug use,and the overuse of antibiotics,has yielded improvements in the safety of China's livestock products.However,some quality and safety concerns persist,leading to varying degrees of risk to public health from meat,eggs,and milk.

In addition,this paper employs a systematic clustering methodology (Q-type cluster analysis) to categorise the level of high-quality development in the livestock industry across provinces[37-38].Based on the systematic clustering analysis results and considering the average level of high-quality development in the livestock industry of each province from 2010 to 2022,the 30 provinces are grouped into four categories: high-quality livestock industry areas,medium-quality livestock industry areas,medium- low quality livestock industry areas,and low-quality livestock industry areas (see Table 3 for results).

Type I:High-quality livestock industry areas.The average level of high-quality development of the livestock sector in 2010-2022 is between 0.25 and 0.31,with only two regions,Jiangsu and Shanghai,included in this category.As provinces renowned for their economic strength,advanced technologies,and robust educational systems in China,their progress in livestock farming can be primarily attributed to a superior environmental friendliness index,comprehensive management of livestock and poultry waste,and higher green total factor productivity.Shanghai,an international metropolis with leading scientific research institutions and a skilled workforce,prioritises environmental protection and has enacted various environmental management measures.These include rigorous measures to reduce air and water pollution alongside active ecological restoration and greening efforts,finally cultivating a greener and more sustainable livestock industry.Jiangsu Province,characterised by its diverse animal husbandry models,centres its approach on scientific and technological advancements,industry upgrades,ecological farming practices,standardised management,and brand developme nt.In addition,the province has implemented a suite of policies promoting livestock industry expansion,including financial subsidies,tax incentives,and scientific and technological support,offering valuable insights for the advancement of animal husbandry in other provinces.

The type II:Medium-quality livestock industry areas.Exhibiting a high-quality development level in the 0.18-0.24 range between 2010 and 2022,this group comprises five provinces:Anhui,Beijing,Fujian,Yunnan,and Chongqing.The moderately high ranking of these five provinces is primarily attributed to their advanced technological and managerial expertise in animal husbandry.While not considered major hubs for livestock production in China,these provinces demonstrate a relatively high level of high-quality development in their respective livestock industries. Anhui and Beijing stand out for the considerable scale of their livestock operations.Fujian exhibits a significant scale level and maintains a significant proportion of breeding livestock and poultry stock nationally.Yunnan and Chongqing,on the other hand,are recognised for their higher levels of mechanisation.Beijing benefits from robust capital,advanced technology,science,and a skilled workforce,resulting in a sophisticated level of animal husbandry development.Pig and poultry farming constitute the backbone of Anhui province's animal husbandry.Influenced by environmental regulations,smaller farms are progressively phased out,paving the path for the expansion of large-scale,modern farming practices,thereby elevating the overall development of animal husbandry in the province.Fujian Province's animal husbandry mainly revolves around poultry production,with a particular emphasis on chicken rearing.This specialization contributes to a lower developmental threshold for the industry.Yunnan Province has a diverse range of livestock species and abundant pastureland,presenting favourable conditions for the advancement of animal husbandry.In recent years,the

Chongqing Municipal Government has implemented a series of supportive policies to cultivate the growth of its livestock industry.Simultaneously,higher requirements for environmental protection and animal welfare have contributed to the steady improvement of high-quality development in the industry.

The type III:Medium- low quality livestock industry areas.This group,with a high-quality development index ranging from 0.14 to 0.18 between 2010 and 2022, includes eleven provinces:Gansu,Guangdong,Guangxi,Guizhou,Hainan,Henan,Heilong jiang,Hubei,Inner Mongolia,Ningxia,and Qinghai.It is worth noting that Heilong jiang,Inner Mongolia,Hubei,and Henan,while being major livestock-producing provinces in China,demonstrate a relatively low level of high-quality development in their livestock industries.This can be attributed to several constraining factors, including underdeveloped livestock infrastructure,cold climatic conditions,a lack of environmental awareness,and a deficiency in high-quality personnel in Heilongjiang.Similarly,Ningxia and Gansu face limitations in achieving a high level of high-quality development in animal husbandry due to factors such as the relative scarcity of water resources.In addition,the average annual growth rate of the high- quality development level in the significant pastoral areas of Inner Mongolia and Qinghai remains low.This slower rate of development reflects the challenges faced in these regions,including issues such as overgrazing,the irrational utilisation of pastureland,arid climatic conditions,and the relative scarcity of water resources.Besides, the persistence of traditional nomadic pastoralism practices in certain areas,combined with a lack of modern breeding methods and management approaches,negatively affects the overall development and effectiven ess of the animal husbandry sector in these regions.

The type IV:Low-quality livestock industry areas.Between 2010 and 2022,these regions,spanning twelve provinces including Hebei, Hunan,Jilin,Jiangxi,Liaoning,Shan dong,Shanxi,Shaanxi,Sichuan,Tianjin,Xinjiang and Zhejiang,exhibited a high-quality development index ranging from 0.09 to 0.14.Specifically,key livestock farming provinces such as Hunan,Shandong,Sichuan,Xinjiang,Hebei,Zhejiang,Liaoning,and Jilin, registered the lowest levels of high-quality development in the national livestock industry.This points to a prevalent issue in Chinese livestock farming:a tendency towards"big production but not good"quality.It is not difficult to find that the southern water network region as an important strategic area of China's pig farming,in addition to Shanghai,Jiangsu and Anhui's higher level of animal husbandry development,the level of animal husbandry development in other regions are generally low,reflecting that pressure for high-quality transformation of the livestock sector remains high in the water network region of southern China.Considering the significant differences in fundamental conditions for animal husbandry development across these provinces, customized approaches to cultivating high-quality advancement are essential.For instance,Shandong Province,a major beef cattle breeding region characterised by extensive farming practices and a high concentration of farms,suffers primarily from environmental pollution.Therefore,future efforts should prioritise the sustainable development of animal husbandry in the region.This can be achieved through a greater emphasis on manure resource utilisation and recycling technologies,alongside the promotion of environmentally sound feed options;whereas,Hunan and Zhejiang,as major pig farming provinces,should focus on optimizing the regional distribution of pig farming operations.This involves enhancing standardised large-scale farming practices and encouraging the establishment of integrated crop-livestock ecosystems that prioritise ecological recycling principles.

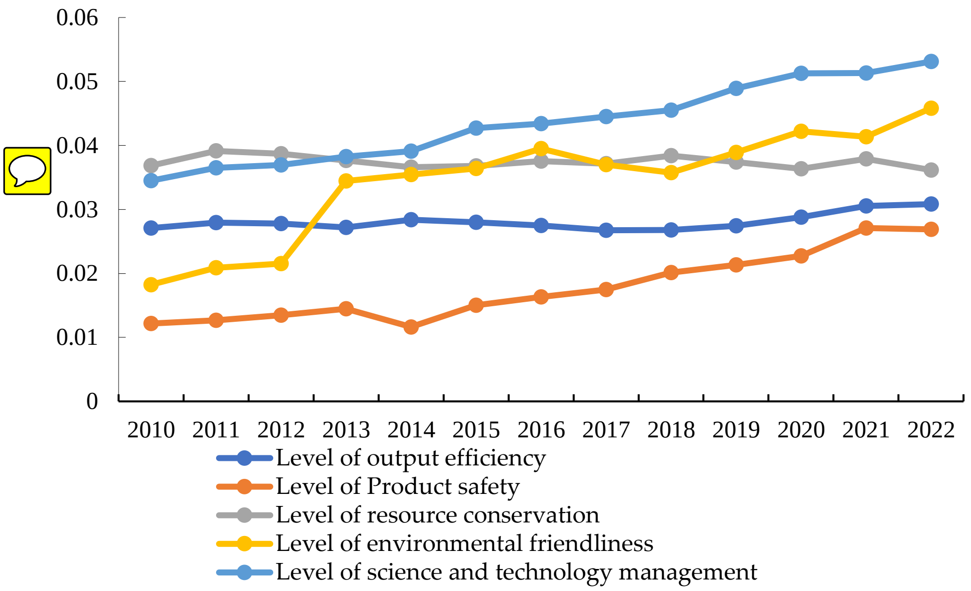

Figure 1. Provincial averages of five dimensions of high-quality development of China's livestock industry,2010-2022

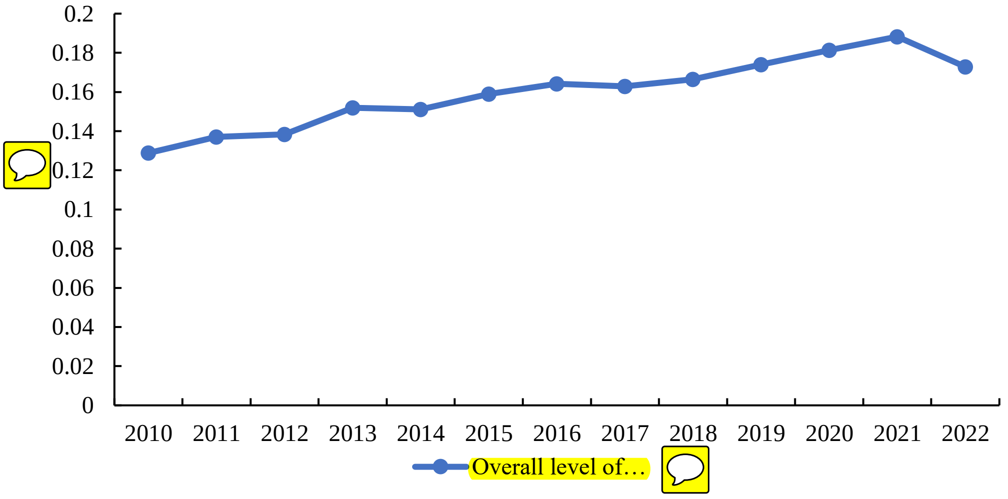

Figure 2. Provincial averages of high quality levels of animal husbandry in China, 2010-2022

Table 2. Level of high-quality development of animal husbandry in 30 provinces in China, 2010-2022

	**2010**	**Rank**	**2012**	**Rank**	**2014**	**Rank**	**2016**	**Rank**	**2018**	**Rank**	**2020**	**Rank**	**2022**	**Rank**	**Annual Growth Rate (%)**
Anhui	0.15	8	0.16	6	0.16	10	0.2	5	0.21	4	0.25	3	0.21	5	2.84
Beijing	0.22	2	0.22	2	0.23	3	0.23	3	0.23	3	0.22	6	0.25	1	1.07
Fujian	0.13	14	0.15	9	0.17	6	0.2	4	0.21	6	0.22	5	0.19	12	3.21
Gansu	0.12	19	0.15	7	0.16	9	0.17	10	0.19	8	0.19	10	0.2	9	4.35
Guangdong	0.15	7	0.15	10	0.16	11	0.17	11	0.17	13	0.18	13	0.21	8	2.84
Guangxi	0.13	12	0.15	11	0.16	12	0.16	13	0.15	18	0.18	14	0.19	11	3.21
Guizhou	0.13	13	0.14	16	0.14	16	0.16	15	0.16	16	0.16	18	0.16	19	1.75
Hainan	0.14	9	0.14	13	0.14	14	0.17	9	0.18	9	0.19	11	0.22	3	3.84
Hebei	0.11	20	0.11	25	0.1	28	0.11	28	0.11	29	0.13	27	0.14	23	2.03

Henan	0.09	29	0.1	27	0.18	5	0.19	7	0.18	10	0.22	7	0.16	18	4.91
Hei-longjiang	0.11	22	0.13	18	0.13	20	0.15	17	0.16	15	0.18	12	0.19	10	4.66
Hubei	0.12	15	0.14	12	0.14	17	0.16	12	0.16	14	0.18	15	0.18	14	3.44
Hunan	0.1	23	0.11	24	0.11	25	0.13	24	0.13	22	0.15	22	0.15	20	3.44
Jilin	0.1	27	0.09	29	0.09	29	0.11	27	0.11	28	0.13	24	0.13	26	2.21
Jiangsu	0.14	10	0.14	14	0.26	2	0.35	2	0.28	2	0.33	2	0.21	6	3.44
Jiangxi	0.1	24	0.1	28	0.11	23	0.12	25	0.12	25	0.15	23	0.13	25	2.21
Liaoning	0.1	26	0.11	23	0.13	21	0.13	20	0.12	24	0.13	26	0.14	24	2.84
Inner Mon-golia	0.16	5	0.16	5	0.15	13	0.16	14	0.16	17	0.16	17	0.18	13	0.99
Ningxia	0.13	11	0.15	8	0.14	15	0.15	16	0.17	12	0.19	9	0.21	7	4.08
Qinghai	0.12	17	0.13	17	0.16	8	0.14	19	0.18	11	0.17	16	0.17	16	2.95
Shandong	0.11	21	0.13	20	0.11	24	0.14	18	0.14	20	0.16	19	0.15	22	2.62
Shanxi	0.07	30	0.09	30	0.08	30	0.08	30	0.1	30	0.12	29	0.13	28	5.29
Shanxi	0.1	25	0.11	22	0.11	26	0.12	26	0.11	26	0.12	28	0.12	29	1.53
Shanghai	0.24	1	0.27	1	0.37	1	0.35	1	0.31	1	0.35	1	0.16	17	-3.32
Sichuan	0.12	16	0.14	15	0.13	19	0.13	21	0.15	19	0.15	20	0.15	21	1.88
Tianjin	0.15	6	0.13	19	0.13	18	0.13	22	0.13	23	0.13	25	0.12	30	-1.84
Xinjiang	0.09	28	0.1	26	0.1	27	0.1	29	0.11	27	0.12	30	0.13	27	3.11
Yunnan	0.16	4	0.16	4	0.17	7	0.18	8	0.2	7	0.22	8	0.22	4	2.69
Zhejiang	0.12	18	0.12	21	0.12	22	0.13	23	0.14	21	0.15	21	0.17	15	2.95
Chongqing	0.16	3	0.17	3	0.19	4	0.19	6	0.21	5	0.24	4	0.22	2	2.69

Table 3. Classification of 30 provinces in China into categories of high quality development level of animal husbandry, 2010-2022

categories	provinces
high-quality areas of the livestock industry(0.25-0.31)	Jiangsu, Shanghai
medium-quality areas of the livestock industry(0.18-0.24)	Anhui, Beijing, Fujian, Yunnan, Chongqing
medium-low quality level of animal husbandry(0.14-0.18)	Gansu, Guangdong, Guangxi, Guizhou, Hainan, Henan, Honglongjiang, Hubei, Inner Mongolia, Ningxia, Qinghai
low-quality areas of animal husbandry(0.09-0.14)	Hebei, Hunan, Jilin, Jiangxi, Liaoning, Shandong, Shanxi, Shanxi, Sichuan, Tianjin, Xinjiang, Zhejiang

3.2. Regional differences in high-quality development of China's animal husbandry industry and their sources

To gain a deeper understanding of the regional differences in the level of high- quality development in China's animal husbandry industry,this study evaluates the overall differences observed between 2010 and 2022.Employing Dagum's Gini coefficient as the primary analytical tool,the research evaluates inter-regional differences,intra- regional

differences,hypervariable density difference and the specific contributions of differences in inter-region differences,intra- region differences,hypervariable density to the overall differences identified.The comprehensive findings derived from these computations are systematically presented in Table 4.

Figure 3 illustrates both the overall differences in the high-quality development of China's animal husbandry industry and its development.The data indicates a pattern of "slow decline-rise-slow decline" in overall regional differences during the observation period.utilizing 2010 as a baseline,regional difference contracted by 1.28% annually.This overall difference steadily decreased from 0.14 in 2010 to 0.13 in 2012,before a slight increase to 0.18 in 2017.Following this, a consistent decline brought it to a nadir of 0.12 in 2022. Broadly,regional differences in the high-quality development of China's livestock industry have remained relatively minor in recent years,fluctuating between 0.12 and 0.18 and exhibiting a marginally downward trend.

Figure 4 illustrates the intra-regional differences and evolutionary trends of high-quality development in the livestock sector across the three primary regions of East, Central,and West.Throughout the observed timeframe (2010-2022),the East exhibited the most significant intra-regional differences,followed by the Central and then the West. Regarding developmental trends,a degree of similarity exists across the three regions. The East demonstrates a pattern of "stable - rapid rise - fluctuating stable - slow decline." Intra-regional discrepancies remained at 0.15 from 2010 to 2012,sharply increased to 0.21 between 2012 and 2013,oscillated between 0.19 and 0.22 from 2014 to 2021,and finally decreased to 0.13 in 2022.Specifically,Shanghai,Jiangsu,Beijing,and Fujian stand out as high to medium-quality development zones in the animal husbandry sector;whereas, Guangdong and Hainan represent areas of medium-low quality,while Hebei,Liaoning, Shandong,Zhejiang,and Tianjin constitute low-quality development areas.This is related to the significant differences among eastern provinces in geographical location,resource availability,policy support,industrial development strategies,and consumer demand.The Central region's development trend reflects this pattern with a"stable-slowly rising- slowly falling."Intra-regional differences in this region held steady at 0.11 from 2010 to 2012,climbed from 0.11 to 0.16 between 2012 and 2017,and fell from 0.16 to 0.10 between 2017 and 2022.Anhui appears to the leading region for animal husbandry development in the Central region.In contrast,Henan,Heilongjiang,and Hubei represent medium-low quality development areas,while Hunan,Jilin,Jiangxi,and Shanxi constitute low-quality zones.Finally,the West demonstrates relative stability in its intra-regional differences, hovering between 0.08 and 0.11.Yunnan and Chongqing have the highest development levels,placing them in the medium-quality tier.Gansu,Guangxi,Guizhou,Inner Mongolia, Ningxia,and Qinghai occupy the medium-low quality tier,whereas Shaanxi,Sichuan,and Xinjiang represent low-quality development areas in the animal husbandry sector.

Figure 5 further illustrates the inter-regional differences and evolutionary trends in the high-quality development of China's livestock industry.The most significant inter- regional differences are observed between the eastern and central regions,exhibiting a pattern of"falling-fluctuating-rising-steady-fluctuating falling."This pattern is characteris ed by a decrease from 0.19 in 2010 to 0.17 in 2012,followed by an increase to 0.22 in 2014.A period of stability ensues from 2014 to 2017,succeeded by a fluctuating decline to 0.13 between 2017 and 2022.Similarly,the eastern and western regions demonstrate a"fluctuating upward-steady-fluctuating downward"trend.This trend comprises a decline from 0.14 in 2010 to 0.13 in 2012,followed by a fluctuating increase to 0.20 in 2016,and a decline to 0.12 in 2022.The smallest difference is identified in the regional difference between the central and western regions,presenting a relatively stable,albeit slightly reducing,trend from 0.14 in 2010 to 0.11 in 2022.At a macro level, the relatively small gap in livestock development between the central and western regions can be attributed to the similar levels of natural resource endowment and socio-economic development in these re-

gions;whereas,the eastern region's significantly advanced overall level of social development,coupled with its unique advantages in animal husbandry,results in its high-quality livestock development being significantly ahead of the central and western regions.

Figure 6 presents the three principal sources of regional difference in the high- quality development of China's livestock industry.The contribution of intra-regional differences to the total variance demonstrates a minor upward trend,from 29.95% in 2010 to 33.09% in 2022;whereas,the contribution rate of inter-regional differences to the total difference exhibits a reducing trend,declining from 47.07% in 2010 to 17.23% in 2022,averaging an annual decrease of 8.03%.This signifies that inter-regional differences exert a waning influence on the overall difference,with 2022 marking their least effect.In contrast,the contribution of hypervariable density differences to the total difference is on the rise,increasing from 22.97% in 2010 to 49.68% in 2022.Overall,the source of regional difference in the high-quality development of the livestock industry has significantly changed,transforming from inter-region > intra-region > hypervariable density in 2010 to hypervariable density > intra-region > inter-region in 2022.The degree of influence exerted by intra-regional differences on the overall differences remains relatively stable.The effect of inter-regional differences on the overall differences gradually leans towards being the least impactful.The degree of influence of hypervariable density on the overall differences gradually tends towards being the most impactful.Specifically, whether in the eastern,central,or western regions,each region comprises provinces exhibiting both relatively high and low levels of high-quality development in animal husbandry.

Table 4. Regional differences in high-quality development of China's livestock sector and sources of differences,2010-2022

		Intra-region			Inter-region			contribution rate(%)		
**Year**	**overall differences**	**eastern**	**central**	**western**	**Eastern-central**	**Eastern-western**	**Central-western**	**Intra-regional**	**Inter-regional**	**hyper-variable**
2010	0.14	0.15	0.11	0.10	0.19	0.14	0.14	29.95	47.07	22.97
2011	0.13	0.15	0.11	0.09	0.18	0.13	0.13	30.11	47.88	22.01
2012	0.13	0.15	0.11	0.08	0.17	0.13	0.13	30.03	38.85	31.12
2013	0.16	0.21	0.13	0.08	0.21	0.18	0.12	31.59	40.22	28.19
2014	0.17	0.22	0.13	0.09	0.22	0.19	0.13	31.45	41.46	27.09
2015	0.17	0.21	0.15	0.09	0.22	0.18	0.13	31.31	37.91	30.78
2016	0.18	0.22	0.15	0.10	0.22	0.20	0.13	31.55	38.32	30.14
2017	0.18	0.22	0.16	0.10	0.22	0.18	0.14	31.78	35.39	32.84
2018	0.16	0.19	0.14	0.10	0.19	0.16	0.13	32.11	29.31	38.58
2019	0.16	0.20	0.14	0.11	0.19	0.18	0.13	32.36	26.46	41.18
2020	0.16	0.20	0.13	0.11	0.18	0.17	0.13	33.02	21.18	45.80
2021	0.15	0.19	0.12	0.10	0.17	0.17	0.12	32.48	22.02	45.50
2022	0.12	0.13	0.10	0.10	0.13	0.12	0.11	33.09	17.23	49.68

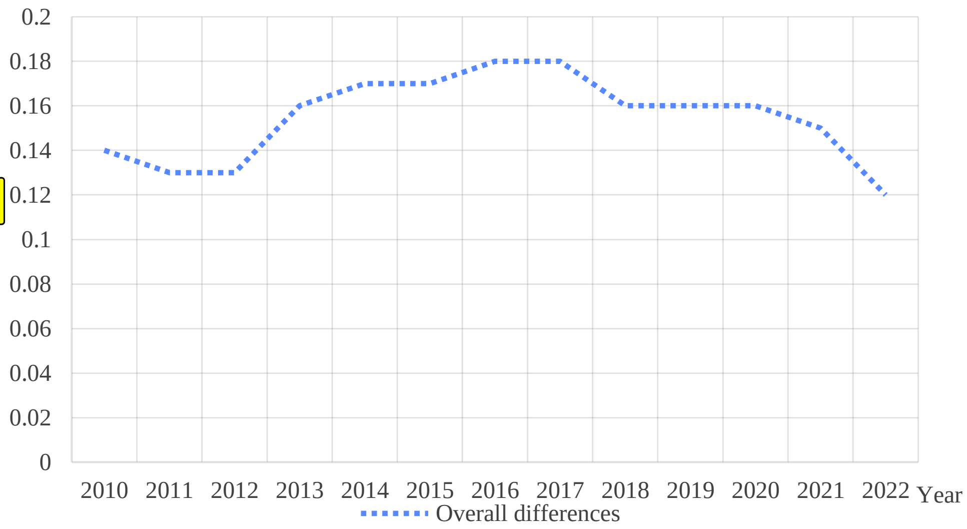

Figure 3. Trends in the evolution of overall differences in the level of high-quality development of China's livestock sector, 2010-2022

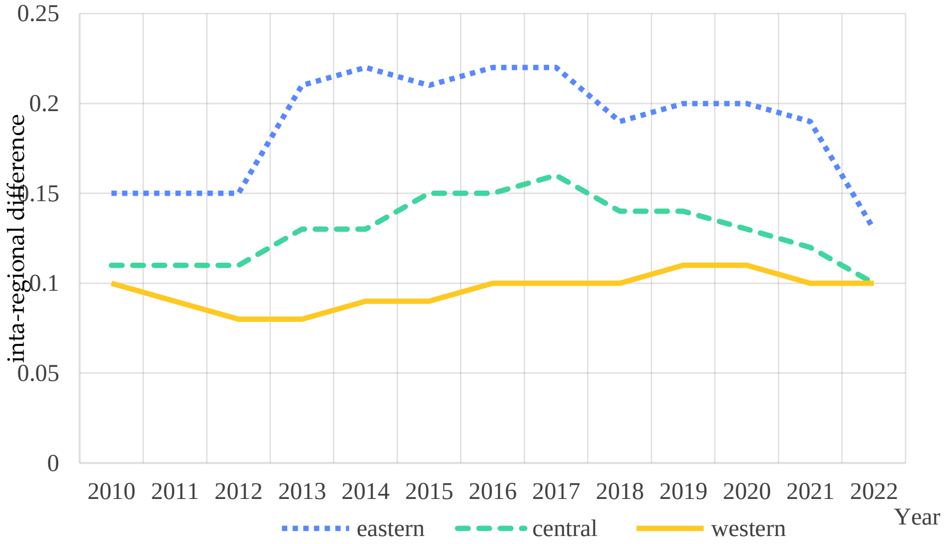

Figure 4. Trends in the evolution of intra-regional differences in the level of high-quality development of China's livestock sector, 2010-2022

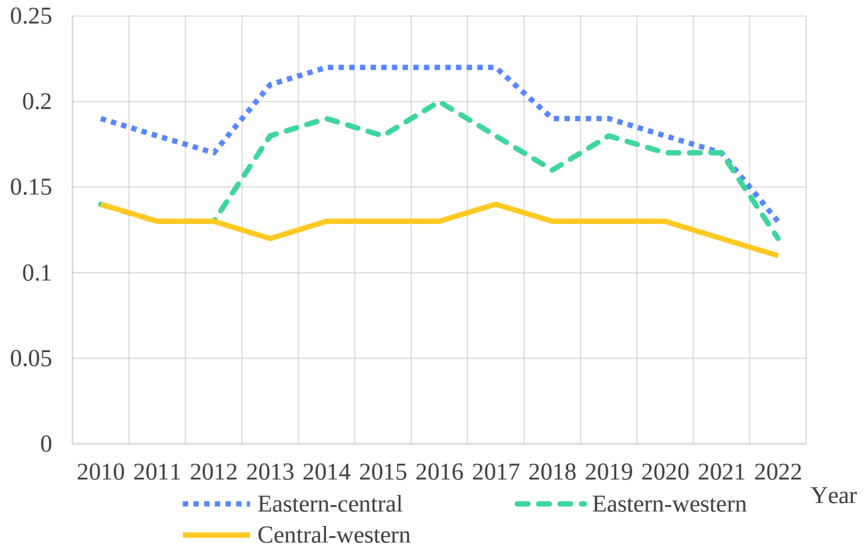

Figure 5. Trends in the evolution of interregional differences in the level of high-quality development of China's livestock sector, 2010-2022

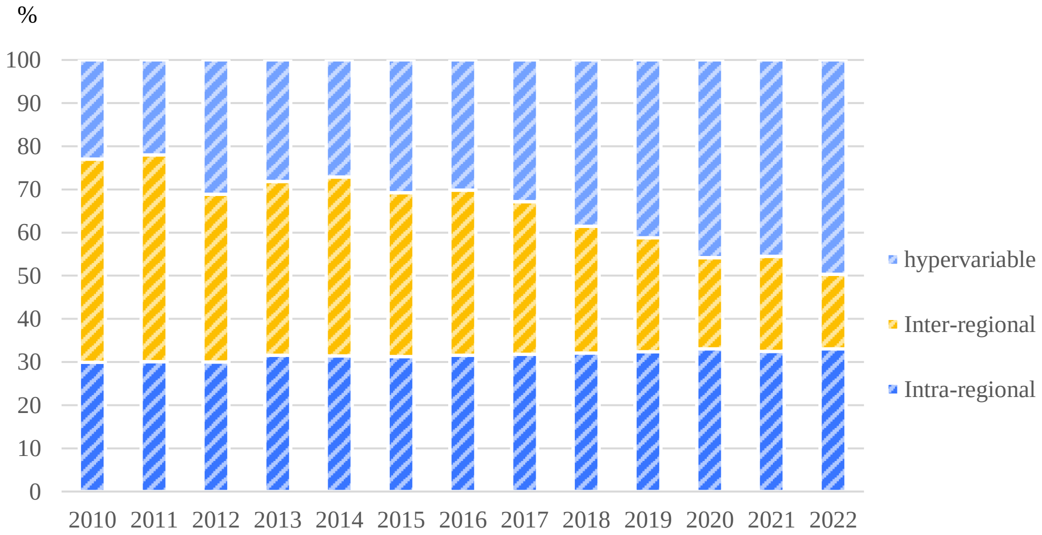

Figure 6. Sources of regional differences in the level of high-quality development of China's livestock sector, 2010-2022

3.3. Characteristics of the temporal dynamic evolution of high-quality development of China's animal husbandry industry

This paper utilises Stata software to map the kernel density of high-quality development in the animal husbandry sector across 30 Chinese provinces over the period 2010 to 2022.Analysis of the distribution dynamics indicate two significant characteristics.Firstly,the central point of the kernel density function,representing high-quality development in the livestock industry,demonstrates a progressive shift towards the right throughout the observed timeframe.In 2012,the high-quality development level of the industry across the 30 provinces was largely concentrated in the 0-0.28 range.However,by 2022,this concentration had shifted to the 0.10-0.40 range. This shift signifies a gradual enhancement in the overall high-quality development level of the livestock industry nationwide.Secondly,the peak of the kernel density curve exhibits a general downward trend,with a twin-peaked curve trend in 2022.This pattern suggests a rising equilibrium

in the high-quality development level of China's animal husbandry industry,accompanied by a certain gradient effect.

The distribution dynamics associated with high-quality development in the eastern region's livestock industry exhibit two main characteristics.Firstly,the kernel density curve,charting the level of high-quality development in the livestock industry,presents with a prominent primary peak and a secondary peak in 2012.The considerable altitude of the primary peak signifies a significant gradient effect,highlighting imbalance in the level of high-quality development across the eastern region's livestock industry in 2012. Secondly,the peak of the kernel density graph in 2012-2022 shows a decreasing and then an increasing trend,while the overarching trend remains downwards.This suggests a narrowing of the differences in high-quality development levels in the eastern region's livestock industry throughout this period,indicating a tendency towards a more concentrated distribution.The distribution range of the kernel density map of the high-quality development level of the livestock industry in the central region is relatively narrow.It shows a rightward trend,with a distribution range of 0.07-0.16 in 2012 and a distribution range of 0.17-0.22 in 2022.The height of the peaks shows a“downward-ascending”trend.This reflects the fact the distribution range of the high-quality development level of the animal husbandry industry in the eastern region is also more concentrated, showing a clear positive trend,and the level of balanced distribution has slightly increased.The kernel density plot charting high-quality development in the western region's livestock industry indicates downward trend in peak height accompanied by a rightward shift in distribution range.This pattern suggests a tendency towards an increased level of high-quality development in the western region's livestock industry,accompanied by an improvement in the overall equilibrium.

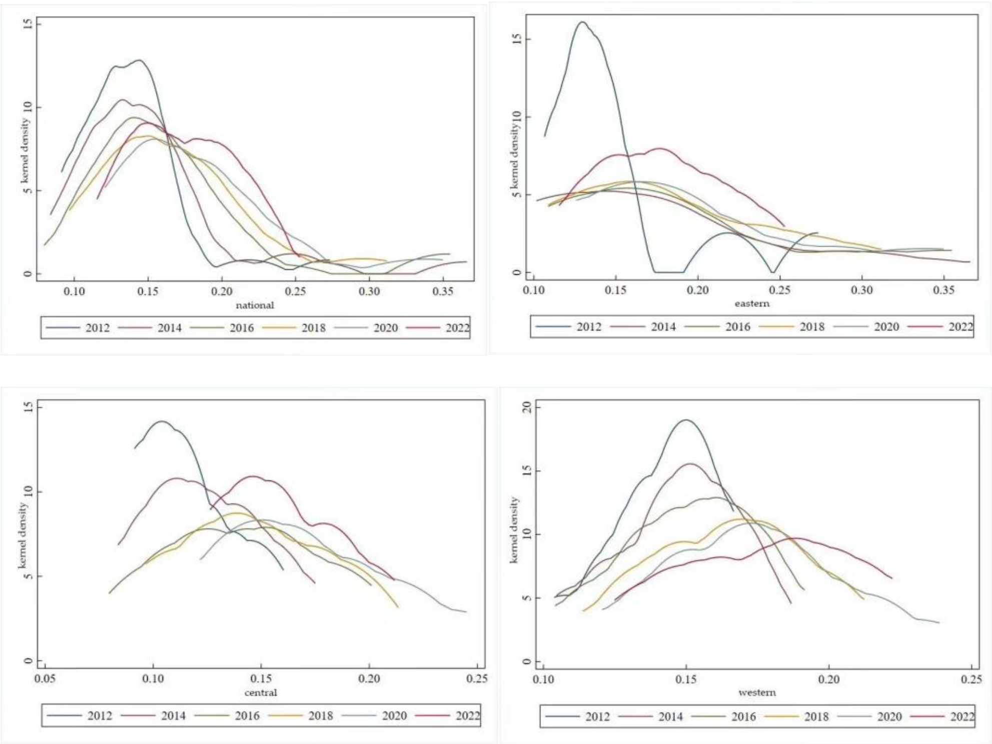

Figure 7. Kernel density of high-quality agricultural development of animal husbandry industry

According to the comprehensive assessment of animal husbandry practices,a four-tier classification system has been established to categorise the level of development across 30 provinces in China.Provinces achieving a comprehensive measurement score in

the interval of [0, 0.1258] are designated as Tier 1, indicating a low level of animal husbandry development.A score in the interval of (0.1258, 0.1510) corresponds to Tier 2,representing a medium-low level of development.Provinces attaining a score in the interval of (0.1510, 0.1808] are classified as Tier 3,signifying a medium level of development.Finally,provinces achieving a score in the interval of (0.1808, +∞) are grouped under Tier 4,indicating a high level of animal husbandry development.

As can be seen from Table 5,the dynamic evolution of the high-quality development level of the livestock industry without considering geospatial factors is characterised as follows:Firstly,the internal mobility among the distribution of high-quality development levels in the livestock industry is limited.The values located diagonally in the low,medium-low,medium,and high-level groups (0.750,0.642,0.724,and0.971,respectivel y)are significantly larger than their non-diagonal counterparts.This suggests a significant "path dependence" and "self-locking" effect in the high-quality development levels of China's various provincial livestock industries.Secondly,the transformation among levels of high-quality development in the livestock industry primarily reflects upward shifts between contiguous subgroups,while the probability of trans-group movements remains insignificant.The low-level group,for instance,presents a 0.250 probability of transitioning to the medium-low level group,with the probability of transitioning to the medium and high-level groups being insignificant.Comparatively,the medium-low level group demonstrates a 0.333 probability of advancing to the medium level group,while the probability of regressing to the low level group is only 0.025,and,again,the probability of reaching the high level group is insignificant. Similarly,the medium level group demonstrates a 0.211 probability of ascending to the high level group,while the probabilities of descending to the medium-low level group (0.053) and the low-level group (0.013)are insignificant.Thirdly, there is a certain degree of "Matthew effect" in the dynamic evolution of the level of high-quality development of the livestock industry.The probabilities of the high-level group and low-level group maintaining their current state are 0.971 and 0.750 respectively,which is significantly higher than the probabilities of the medium-level group (0.724) and medium-low level group (0.642) maintaining their current states.That is to say,it is very easy for the high level group area to keep the leading state,but it is difficult for the low level group area to get rid of the lagging state.

The transfer probability in the spatial Markov chain exhibits more significant differences compared to the traditional Markov chain model.For instance,when the effect of neighbouring areas is not factored in,the probability of a low-level group transitioning to a low-middle level group stands at 0.250.However, when a low-level group is spatially adjacent to low-level,medium-low level,medium-level,and high-level group areas,the probabilities associated with this upward transition are 0.106,0.333, 0.857,and 0.333,respectively.This finding indicates that the probability of an upward transition for a low-level group decreases when bordered by other areas of similar, low-level status;whereas,the potential for upward transition is significantly greater when these low-level groups border medium-low level,medium-level,and high-level groups,with the probability reaching its peak when adjacent to a medium-level group. When the spatial influence of neighbouring areas is not considered,the upward transition probability for medium-low level groups is calculated to be 0.333.This probability is significantly changed to 0.250,0.192,0.406,and 0.545 when bordering low- level,medium-low level,medium-level,and high-level groups,respectively.This analytical incorporation of the spatial factor indicates that the proximity to both low and medium-low level groups negatively affect the probability of upward transition for medium-low level groups, causing a decrease.This probability only exhibits an increase when bordering medium and high-level groups,reaching its highest point when adjacent to a high-level group.For areas categorically classified as medium-level,the probability of experiencing an upward transition is 0.211 when the effect of neighbouring areas is disregarded.This probability changes,becoming 0.250, 0.167, 0.188, and 0.250 when bordering low-level,medium-low level,me-

dium-level,and high-level groups,respectively. It worth noting that the probability of upward transition for the medium-level group displays only a marginal increase when it neighbours a high-level group.For a high-level group,the probability of remaining stably in the same group is almost 1 when adjacent to low-level,medium-low level,medium-level,or high-level groups.This observed trend reflects the fact that the development of the livestock industry in high-level groups is highly stable and exhibits characteristics of path dependency,with the probability of a downward transition being highly insignificant.

Table 5. Markov chain transfer distribution for high-quality development of China's livestock industry

Space Lag Type	t/t+1	low level	medium-low level	medium level	high level	n
Traditional	low level	0.750	0.250	0.000	0.000	84
	medium-low level	0.025	0.642	0.333	0.000	81
	medium level	0.013	0.053	0.724	0.211	76
	high level	0.000	0.000	0.029	0.971	69
low level	low level	0.894	0.106	0.000	0.000	47
	medium-low level	0.000	0.750	0.250	0.000	12
	medium level	0.000	0.000	0.750	0.250	4
	high level	0.000	0.000	0.000	1.000	0
medium-low level	low level	0.667	0.333	0.000	0.000	27
	medium-low level	0.000	0.808	0.192	0.000	26
	medium level	0.000	0.083	0.750	0.167	12
	high level	0.000	0.000	0.000	1.000	11
medium level	low level	0.143	0.857	0.000	0.000	7
	medium-low level	0.063	0.531	0.406	0.000	32
	medium level	0.031	0.063	0.719	0.188	32
	high level	0.000	0.000	0.000	1.000	20
high level	low level	0.667	0.333	0.000	0.000	3
	medium-low level	0.000	0.455	0.545	0.000	11
	medium level	0.000	0.036	0.714	0.250	28
	high level	0.000	0.000	0.053	0.947	38

3.4 Characteristics of the spatial correlation of the high-quality development of China's animal husbandry industry

Based on the modified gravity model,this paper calculates the strength of correlation of high-quality development of animal husbandry among provinces,and uses social network analysis to portray the characteristics of the network structure and the evolution trend of China's high-quality development level of animal husbandry from 2010-2022.Table 6 shows the spatial network density and correlation of high-quality development of China's livestock sector from 2010 to 2022.

Network density is the ratio between the number of links that actually exist in the network and the theoretical maximum number of links that can exist,which is used to measure the closeness and complexity of the network[39].The number of links between provinces in the high-quality development of animal husbandry network has increased from 177 in 2010 to 189 in 2022.The density of the network remains at a low level between 0.20 and 0.22,but shows a slight increasing trend,indicating that the degree of linkage of the high-quality development level of the livestock industry between provinces shows a trend of tightening and complexity,but is still at a low level.The mobility of the relevant

production factors and resources in each province is weak,and the degree of spatial association of the high-quality development level of animal husbandry industry between provinces still has a large room for improvement,and the efficient reorganisation and allocation of interregional resource factors is expected to be further strengthened.The connectedness degree[40] has been 1 during the sample period, indicating that there are no isolated provinces in the high-quality development level of China's animal husbandry industry and all provinces have established certain links with other provinces.The degree of network hierarchy[41] has always remained at a high level, rising from 0.59 in 2010 to 0.71 in 2022,reflecting the fact that the level of high-quality development of China's animal husbandry industry shows obvious hierarchical characteristics,which are becoming more and more strict.The degree of differentiation in the level of high-quality development of China's animal husbandry industry is also becoming more and more obvious,and the "spatial spillover effect" and "diffusion effect" need to be further strengthened.Network efficiency remains at a high level between 0.67 and 0.71 and shows a slight downward trend,reflecting the existence of certain redundant relationships and multiple superimposed spillover channels in the level of high-quality development of China's animal husbandry industry.

In terms of the outdegree[42],the outdegree of each province remained between 1-9 in 2010 and between 2-10 in 2022,showing a stable trend,reflecting that the spatial spillover effects and diffusion effects of China's high-quality development of animal husbandry did not show a trend of intensification in the period from 2010 to 2022.In terms of specific provinces,Fujian,Guizhou,Shanghai,Henan,Hunan, Gansu,Guangdong, Heilongjiang,Ningxia,Qinghai,Sichuan,Xinjiang,Yunnan,Chongqing,Hubei and Guangxi remain above seven provinces,which are among the provinces with higher point outdegree.It is not difficult to find that the provinces with high point outdegree are mainly the main producing provinces of China's livestock industry,even though the level of high-quality development of the livestock industry in these provinces is not very high.Hainan, Jiangsu,Jiangxi,Jilin,Shandong,Shanxi,Shanxi,and Zhejiang are in a medium level state in terms of the spillover effect of the level of high-quality development of the animal husbandry industry.Jiangsu,which has a good level of high-quality development of animal husbandry,has insufficient spatial radiation-driven capacity for high-quality development of the livestock industry in the surrounding areas.Anhui,Hebei,Liaoning, Inner Mongolia,Tianjin,Beijing's point outdegree is in the 4 or less,the spatial spillover ability to remain in the bottom level state.

In terms of the indegree[42],the average point indegree of each province has increased from 5 in 2010 to 6 in 2022.The point indegree of Shanghai,Beijing, Jiangsu, Zhejiang has always been in the top four,maintained in more than 20 provinces, and the point indegree is significantly larger than the point outdegree, reflecting that the"Siphon effect" of the high-quality development of animal husbandry in Shanghai,Beijing,Jiangsu, Zhejiang is larger than the"trickle-down effect".Guangdong and Tianjin,whose point indegree is maintained at around 10 provinces,also have a strong"Siphon effect".Hainan, Heilongjiang,Jilin,Liaoning,Inner Mongolia,Ningxia,Gansu,Yunnan,Xinjiang,Shanxi, Shanxi,and Sichuan have almost 0.Combined with the point indegree,it can be found that the point outdegree of the main livestock-producing provinces is generally greater than the point indegree,i.e.,the"trickle-down effect"is greater than the "Siphon effect",so it is evident that the main livestock-producing provinces are exporting more resources than they are attracting.The main reason for this is that on the one hand,the major livestock-producing provinces need to constantly export livestock products to other provinces,on the other hand,due to the relatively low degree of concentration in the livestock industry and the lack of technological innovation and other reasons that lead to the low productivity and economic efficiency of the major livestock-producing provinces, and are unable to attract a large influx of human capital and other factors.Shanghai, Beijing,Jiangsu and Zhejiang are the most economically and socially developed provinces in China,thus attracting a large influx of talent,capital,technology and other resources into the region.

The Closeness centrality[43]can reflect the extent to which the high development of animal husbandry in a province is not controlled by other regions.On average,the closeness index of each province grows from 12.92 in 2010 to 20.06 in 2022,reflecting the increasing independence of the level of high-quality development of China's animal husbandry industry in each province and the increasing ability to control resources.The closeness indexes of Beijing,Shanghai,Tianjin,Jiangsu,Zhejiang and Guangdong are always in the top ten,reflecting the their low degree of external dependence and their ability to rely on the resources of their own provinces to achieve high-quality development of the livestock industry.The closeness index of Ningxia,Qinghai,Gansu, Heilongjiang,Shanxi, Yunnan,Xinjiang,Hainan,Inner Mongolia,Jilin,Liaoning and Shanxi is always below 10,reflecting that the main livestock-producing provinces in China have a weak independence of livestock development,have little control over resources and information,and need the support of other provinces in terms of technological innovation,human capital,and management experience in the process of promoting high-quality development of the animal husbandry industry.

The degree of Betweenness centrality[43] can reflect the ability of each province to control the resource elements in the network of high-quality development of the livestock industry.In 2010 and 2020,the betweenness centrality of Guangdong,Shanghai, Guizhou,Henan,Jiangsu and Jiangxi are consistently in the top ten,reflecting the fact that these ten provinces have a certain degree of control over the resource elements of the national animal husbandry development.The betweenness centrality of Shandong, Zhejiang,Tianjin and Hebei shows a clear downward trend,reflecting that the control of these five provinces over the resources of animal husbandry tends to decline.The betweenness centrality degree of Fujian shows a clear upward trend,reflecting the reinforcement of Fujian's control over resources and information in the high-quality network linkages of the animal husbandry industry.The betweenness centrality of Hunan,Hubei,Beijing,Sichuan,Gansu,Heilongjiang,Jilin,Liaoning,Ningxia,Qinghai,Hainan,Xinjiang,Inner Mongolia,Shanxi,Shaanxi,and Yunnan are in a state of very low level,staying below 1,reflecting once again the weak control of resource elements in China's main farming producing regions.The main farming provinces have not yet developed animal husbandry into an absolutely dominant industry,market competitiveness is weak,and the phenomenon of resource outflow is obvious.The deep- seated reason lies in the fact that China's livestock farming is still a decentralised operation,with a short industrial chain and low economic benefits for farmers,who are unable to give full play to the production benefits brought about by the agglomeration of resources.

Table 6. Characteristics of spatial correlation network for high-quality development of China's animal husbandry industry

Year	number of associations	Density	Correlation analysis		
			Connectedness	Hierarchy	Efficiency
2010	177	0.20	1	0.59	0.71
2011	181	0.21	1	0.55	0.70
2012	184	0.21	1	0.59	0.69
2013	190	0.22	1	0.59	0.68
2014	191	0.22	1	0.72	0.68
2015	193	0.22	1	0.62	0.67
2016	190	0.22	1	0.65	0.68
2017	188	0.22	1	0.68	0.68
2018	183	0.21	1	0.71	0.70
2019	187	0.21	1	0.71	0.69

2020	179	0.21	1	0.71	0.70
2021	184	0.21	1	0.71	0.69
2022	189	0.22	1	0.71	0.68

Table 7. Spatial correlation network centrality analysis of high quality development of animal husbandry in Chinese provinces

	OutDegree	InDegree	Closeness	Betweenness	OutDegree	InDegree	inCloseness	Betweenness
Anhui	4(6)	1(12)	29.95（9）	0.53(16)	4(8)	4(10)	14.08(12)	0.2(18)
Beijing	1(9)	1(12)	45.93（1）	1.19(14)	2(9)	25(2)	45.73(1)	2.37(9)
Fujian	8(2)	24(2)	22.78（14）	1.32(12)	8(4)	15(4)	14.64(7)	8.43(1)
Gansu	7(3)	1(12)	5.72(22)	0.03(19)	10(1)	2(12)	6.21(21)	0.92(13)
Guangdong	8(2)	6(8)	30.10（7）	15.70(1)	9(2)	11(5)	14.26(10)	3.85(6)
Guangxi	6(4)	4(10)	21.60（15）	0.36(17)	9(3)	3(11)	13.12(16)	2.28(10)
Guizhou	9（1）	6(8)	21.44（16）	7.18(3)	9(3)	4(10)	13.18(15)	3.13(8)
Hainan	6(4)	0(13)	5.65(24)	0(20)	6(6)	0(14)	5.65(26)	0(22)
Hebei	2(8)	21(4)	29.30（10）	2.26(11)	2(9)	7(7)	30.22(3)	0.34(16)
Henan	7(3)	15(5)	30.07（8）	6.43(4)	7(5)	8(6)	14.54(8)	5.97(4)
Heilongjiang	7(3)	2(11)	5.88(20)	0(20)	8(4)	0(14)	6.01(22)	0(22)
Hubei	5(5)	26(1)	27.85（12）	1.20(13)	7(5)	7(7)	13.74(13)	1.69(12)
Hunan	6(4)	24(3)	25.34（13）	1.17(15)	7(5)	6(8)	13.26(14)	0.4(14)
Jilin	7(3)	2(11)	5.88(21)	0(20)	6(6)	0(14)	5.52(28)	0(22)
Jiangsu	6(4)	5(9)	42.85（3）	6.27(6)	6(6)	25(2)	16.05(5)	6.93(3)
Jiangxi	6(4)	2(11)	28.28（11）	4.42(8)	6(6)	5(9)	14.28(9)	5.08(5)
Liaoning	4(6)	14(6)	5.64(28)	0(20)	4(8)	0(14)	5.50(29)	0(22)
Inner Mongolia	4(6)	0(13)	5.52(30)	0(20)	4(8)	1(13)	5.56(27)	0.02(21)
Ningxia	7(3)	0(13)	6.00（19）	0(20)	8(4)	0(14)	6.45(19)	0(22)
Qinghai	7(3)	4(10)	5.66(23)	0(20)	8(4)	0(14)	6.44(20)	0(22)
Shandong	7(3)	6(8)	32.66（5）	6.31(5)	5(7)	5(9)	14.16(11)	0.38(15)
Shanxi	6(4)	0(13)	5.55(29)	0(20)	4(8)	0(14)	5.50(30)	0(22)
Shanxi	6(4)	0(13)	5.65(25)	0(20)	5(7)	2(12)	5.77(23)	0.09(20)
Shanghai	7(3)	0(13)	45.11（2）	8.08(2)	7(5)	26(1)	16.17(4)	7.83(2)
Sichuan	6(4)	0(13)	14.38（18）	0.14(18)	8(4)	1(13)	11.23(18)	0.1(19)
Tianjin	3(7)	0(13)	31.42（6）	4.38(9)	2(9)	11(5)	31.38(2)	0.22(17)
Xinjiang	6(4)	9(7)	5.65(26)	0(20)	7(5)	0(14)	5.66(25)	0(22)

Yunnan	6(4)	0(13)	5.65(27)	0(20)	8(4)	0(14)	5.72(24)	0(22)
Zhejiang	6(4)	0(13)	37.38（4）	6.04(7)	5(7)	16(3)	15.15(6)	1.89(11)
Chongqing	7(3)	4(10)	16.93（17）	3.46(10)	8(4)	5(9)	12.34(17)	3.54(7)

Note:Rankings of provinces are in parentheses

3.5. Analysis of factors affecting the high-quality development of China's livestock industry

The previous study measured the level of high-quality development in China's livestock industry and analysed its spatiotemporal evolutionary trend. It is necessary to conduct a deeper analysis of the factors influencing this development to offer insights of how to cultivate high-quality development in the Chinese livestock industry. This paper evaluates these influencing factors from a macro perspective,employing the following analytical model:

$$LI_{it} = \alpha_0 + \alpha_1 PGDP_{it} + \alpha_2 URB_{it} + \alpha_3 TEC_{it} + \alpha_4 TRA_{it} + \alpha_5 IND_{it} + \alpha_6 FIN_{it} + \alpha_7 ARI_{it} + \alpha_8 POD_{it} + \alpha_9 PFA_{it} + \gamma_i + \mu_t + \rho_{it}$$

The left-hand side of the equation represents the level of high-quality development in the livestock sector in province i in year t.Among the explanatory variables on the right-hand side of the equation,PGDP represents GDP per capita,while URB represents the urbanisation rate.In addition,TEC denotes the level of scientific and technological development in the livestock industry,measured by the sum of agricultural patents due to the absence of separate data specifically for the livestock industry.TRA signifies the level of transport,measured by the transport network density in each province and calculated as the ratio of the total number of miles of railways and highways to the area of the administrative divisions of each province.IND represents the industrial structure, measured by the proportion of primary industry in the total sum of primary,secondary, and tertiary industries.FIN denotes the level of agricultural financial development, expressed by the total amount of loans for agriculture,forestry,animal husbandry,and fishery in each province.ARI signifies the structure of the agricultural industry, expressed as the proportion of animal husbandry output value in the total output value of agriculture.POD represents population density,measured by the ratio of the year-end resident population to the area of the administrative divisions in each province at the year's end.Finally,PFA denotes the level of fiscal support for agriculture,expressed as the percentage of local fiscal expenditure on agriculture,forestry,and water affairs in the fiscal general budget expenditures.The variables $\alpha_1 \sim \alpha_6$ are the coefficients to be estimated for each of the influencing factors,while, α_0 、 γ_i 、 μ_t and ρ_{it} refer to the intercept term,individual effect,time effect,and random interference term,respectively.The data in this section are primarily sourced from the China Statistical Yearbook and the statistical yearbooks of each province.

Table 8 reports the regression results of the factors influencing the level of high-quality development in China's livestock sector.To ensure robust findings,this paper employs six regression methods.The initial column displays the results of the panel mixed least squares regression utilising clustered robust standard errors,while the second column presents the results of the fixed effects regression,also employing clustered robust standard errors.The third column details the results of the two-way fixed effects regression,controlling for both time and individual variables.The fourth column indicates the results of the random effects regression utilizing clustered robust standard errors.Specifically,both panel fixed-effects and random-effects models operate under the assumption of homoskedasticity and an absence of autocorrelation.Should heteroskedasticity and autocorrelation be present in the model,pseudo-regression may arise.This paper utilises both the White test and the BG test to appraise the presence of heteroskedasticity and autocorrelation in

the model,respectively.With both tests rejecting the original hypothesis,the model constructed in this paper significantly exhibits both heteroskedasticity and autocorrelation.Drawing upon the research conducted by Lei Zhao and Cheng Fang (2019),two treatment methods are employed. Firstly,regression utilizing Feasible Generalised Least Squares (FGLS) is implemented, followed by the correction of the standard deviation of the Ordinary Least Squares (OLS) model regression utilizing the panel-corrected standard deviation (PCSE).The results of these regressions are presented in columns five and six of Table 6, respectively [44-45].

Regression analysis indicates that the level of high-quality development in China's livestock industry is influenced by a multitude of factors.Most specifically,the rate of urbanisation demonstrates a significant positive correlation with this development, indicating that increased urbanisation actively cultivates improvement in the industry. This phenomenon can be attributed to several key factors.Firstly,rising urbanisation rates often lead to an increase in non-agricultural employment opportunities for farmers, contributing to a general upward trend in societal income levels.Therefore,demand for livestock products experiences gradual growth,further stimulating the development of animal husbandry practices.Moreover,increased urbanisation facilitates improvements in human capital,attracting more skilled individuals to the field and thereby cultivating advancements in scientific and technological progress,as well as elevating management standards in the industry.In addition,a greater level of urbanisation inevitably affects the livestock industry's mode of production,leading to a decline in small-scale family farms and encouraging a shift towards larger-scale operations characterised by specialisation and modernisation.In conclusion,the increase of urbanisation in China significantly generates far-reaching and positive consequences for the high-quality development of the nation's animal husbandry industry.

The extent of scientific and technological advancement has a significantly positive effect on the progress of the livestock industry,that is to say,developments in these areas can precipitate the evolution of the industry towards higher quality operations.Scientific and technological innovation should be regarded as the driving force behind such progress.Considering finite resources,it is a vital tool with which to optimise feed composition,maximise the use of waste products,optimize breeding programmes for superior livestock,and develop more sophisticated approaches to disease prevention and control.These factors,accordingly,lead to improved production efficiency,enhanced product quality and greater environmental sensitivity,all of which contribute to a more sustainable future for the livestock industry.

The quality of transport infrastructure significantly affects the progress of the livestock industry towards high-quality development.Improved transport infrastructure is conducive to optimising the circulation and accessibility of livestock products in the market.This optimisation reduces transport costs while simultaneously preserving the freshness and quality of livestock products.Moreover,it facilitates a more efficient and reliable system for the supply of essential production factors,such as feed and manufacturing equipment,required by livestock breeders.The enhancement of transport infrastructure also strengthens the livestock industry chain by enhancing processing, storage,and transport capabilities for livestock products.Such improvements contribute to the increased value of livestock products and further extend the industry chain. Simultaneously,improvements in transport infrastructure can propel the progress of cold chain logistics and e-commerce platforms for livestock products,thereby expanding both sales channels and market reach.

The share of the agricultural industry in the total industry can have a positive impact on the high quality of the livestock sector.On the one hand,as the agricultural industry expands,the cultivation and supply of feed crop will also increase,which will help to increase the source of forage for the livestock industry and reduce forage costs,thereby promoting the high-quality development of the livestock industry.The development of the agricultural industry is also conducive to the combination of agriculture and livestock and

recycling.The development of the agricultural industry can provide more agricultural and sideline products,which can be used as feed for the livestock industry or as raw materials in other areas,thus promoting the combination of agriculture and livestock and the recycling of resources,thereby improving the efficiency and sustainability of the livestock industry.On the other hand,the development of agricultural industry is usually accompanied by technical support and industrial coordination.Technological progress and industrial coordination can promote the synergistic development of agriculture and animal husbandry,forming a virtuous circle and promoting the development of animal husbandry to high quality.

The extent of agricultural financial development significantly contributes to the high-quality development of the livestock industry:greater loan provision for agriculture, forestry,animal husbandry,and fisheries directly correlates with advancements in the livestock husbandry.A sophisticated agricultural financial system,equipped to offer more financial support to the livestock industry,is also better positioned to offer insurance products and risk management strategies,enhancing the industry's resilience against unforeseen events such as natural disasters and epidemics.In addition, environmentally-focused financial instruments,including green credit and carbon finance,can steer animal husbandry production towards sustainable practices that conserve resources and protect the environment.Finally,widespread access to financial services,particularly for smallholder farmers,can facilitate the modernisation of livestock production methods, finally driving high-quality development throughout the industry.

The agricultural industry's structure significantly affects the high-quality develop ment of the livestock industry.Specifically,a greater proportion of livestock industry output value in the overall agricultural output value positively correlates with the optimisation of the livestock industry.A higher proportional value signifies that livestock production holds greater significance in the agricultural sector and experiences more robust consumer demand.This results in the allocation of increased resources (e.g.,water resources,land resources,feedstuffs,etc.),towards the livestock sector's development.Moreover,this significance attracts scientific research organisations, technological support,and innovative resources to be invested in animal husbandry. Furthermore,governmental bodies are likely to introduce more supportive policies and funding to steer the livestock industry towards a more efficient and sustainable model.

A certain correlation exists between population density and the level of high-quality development in the livestock industry:a higher population density suggests conditions more conducive to such development.Theoretically,a densely populated region implies a more extensive consumer market,yielding greater economic advantages for those engaged in animal husbandry.In addition,areas with high population density often exhibit a greater concentration of research facilities,technical expertise,and resources for innovation,cultivating a more fertile ground for technological advancement in animal husbandry.Livestock farming in such regions may also have a greater capacity to attract investment and technical assistance,thereby promoting more efficient and sustainable practices.In conclusion,a higher population density generally signifies greater regional strength,which strengthens the capacity for high-quality development in the livestock industry.

Table 8. Regression results of factors influencing the high-quality development of China's animal husbandry industry

Variables	(1)	(2)	(3)	(4)	(5)	(6)
	OLS_robust	FE_robust	FE_TW	RE_robust	FGLS	PCSE
PGDP	0.111***	0.013	0.00576	0.00957	-0.0246***	0.00522
	-0.0312	-0.02	0.0202	-0.025	-0.00923	-0.029
URB	-0.0683	0.152**	0.326***	0.160***	0.352***	0.338***

	-0.0569	-0.0639	0.0856	-0.0543	-0.0285	-0.0628
TEC	0.0392	0.0393*	0.0250***	0.0370*	0.0189***	0.0284***
	-0.0253	-0.0197	0.00806	-0.0191	-0.00346	-0.00579
TRA	0.0189	0.0313**	0.0263*	0.0263*	0.0182*	0.0281***
	-0.0175	-0.0147	(0.0146)	-0.0157	-0.00967	-0.00846
IND	0.217*	0.221	0.191*	0.220***	0.0487	0.202***
	-0.114	-0.134	(0.114)	-0.0827	-0.0974	-0.0436
FIN	-0.00654	0.00665	0.00658**	0.00691*	0.00185*	0.00649***
	-0.00522	-0.00422	(0.00262)	-0.00365	-0.00099	-0.00246
ARI	0.0447	0.133***	0.0878**	0.0820**	0.105***	0.0946***
	-0.0609	-0.0382	(0.0383)	-0.0378	-0.0271	-0.0288
POD	0.0380***	0.0822	0.217***	0.0300***	0.168***	0.236**
	-0.0104	-0.124	(0.0662)	-0.0111	-0.0377	-0.109
PFA	0.531***	0.0284	0.00725	0.135	0.102	0.0436
	-0.187	-0.092	(0.104)	-0.0942	-0.0691	-0.0512
Constant	0.0044	-0.0862	-0.216***	-0.0596*	-0.139***	4.144
	-0.0429	-0.063	(0.0639)	-0.0307	-0.0282	-3.371
Observations	369	369	390	390	390	390
R-squared	0.572	0.567	0.515	0.479	——	0.8734

In order to further examine the regional heterogeneity of factors affecting the high-quality development of China's livestock industry,regressions were conducted on the factors affecting the high-quality development of the livestock industry in the eastern,central,western,and north-eastern regions,respectively.The results are shown in Table 9.

(1) GDP per capita has had a positive impact on the high quality development of the livestock sector in the eastern and western regions.However,for the central region,the rapid economic development has inhibited the high-quality development of the livestock industry,possibly because with rapid economic development,the demand for water,land and other resources in the central region increased and a large amount of land was used for urban and industrialised construction,triggering a more serious competition for resources,which led to the livestock industry facing a serious shortage of water and land resources and restrict the high-quality development of the livestock industry.GDP per capita has no impact on the high quality of livestock farming in the Northeast,which is inevitably related to the relatively low level of economic development in the Northeast. (2) The urbanisation rate had a significant positive impact on the high quality development of the livestock sector in the eastern and western regions and had no impact on the high quality development of the livestock sector in the central and north-eastern regions.On the one hand,compared with the central and northeastern regions,the urbanisation process in the eastern and western regions is faster,and the residents'demand for high-quality,green and safe livestock products is increasing rapidly, which stimulates the high-quality development of the livestock industry.On the other hand,the rapid development of urbanisation in the eastern and western regions has accelerated the rhythm of farmers going to the cities to work,which is conducive to promoting the transformation of the production mode of the animal husbandry industry from the decentralised operation of small farmers to the direction of scaling up and industrialisation, and is also conducive to the introduction and adoption of advanced technology. (3) The level of scientific and technological development has a significant positive impact on the high-quality development of animal husbandry in the east,west,centre and north-east of China. (4) The improved level of transport has had some positive impact on the eastern and central regions, some negative impact on the north-eastern region and no impact on the

western region.The likely reason for this is that the good transport network in the eastern and central regions provides convenient marketing channels for livestock products,promotes the circulation and allocation of factors of production,and improves transport efficiency and profitability,which in turn promotes high-quality development of the livestock industry.The northeastern region is relatively resource-poor,and the improved level of transport promotes the flow of resources to developed regions,inhibiting the high-quality development of the livestock industry in the northeastern region.The foundation for the development of the livestock industry in the western region is relatively weak,and even if the level of transport is raised,it is difficult to achieve significant development due to factors such as infrastructure and resource endowment. (5) The industrial structure affects the east and west,i.e.the higher the share of agriculture in the total industry,the more favourable it is for the high quality development of the livestock sector. In contrast,the industrial structure has a non-significant impact on the centre and the north-east. The central and northeastern regions are the main agricultural production areas in China,but the agricultural production methods are still plagued with problems such as low production efficiency and serious waste of resources.Therefore,even if the value of agricultural output is increasing, it does not mean that the level of high-quality development of agriculture is raising. (6) There is a significant positive effect of the level of agricultural financial development on the high quality development of the livestock sector in the eastern, western and north-eastern regions.The impact on the level of high-quality development of the livestock industry in the central region is not significant,the possible reason is that agricultural finance in the central region is more oriented towards the plantation industry and has less coverage of the livestock sector.The central region should promote more financial products and service support for the high-quality development of the livestock industry,and formulate policies to attract more funds to invest in the high-quality construction of the livestock industry. (7) The structure of the agricultural industry has a significant positive impact on the development of high quality in the livestock industry in the western and central regions, and has no impact on the northeastern region,possibly because of the relative scarcity of water and land resources in the eastern region, and a high proportion of the value of animal husbandry output will exacerbate the resource constraints and limit the high quality of animal husbandry development. (8)Population density has a positive effect on the high-quality development of the livestock industry in the east and west regions,and does not have a significant effect on the high-quality development of the livestock industry in the central and north-eastern regions.The possible reason for this is that the eastern and western regions are more densely populated,and the market demand is relatively strong,which stimulates the high-quality development of the livestock industry.At the same time,high population density is conducive to the centralised allocation and utilisation of resources,as well as technological innovation and experience exchange,thus positively affecting the high-quality development of the livestock industry in the eastern and western regions.In contrast,the relatively low population density in the west and north-east has yet to play a role in promoting the high-quality development of the livestock industry. (9)Fiscal support for agriculture will have some impact on the western and north-eastern regions and no impact on the eastern and central regions.Shanxi and Henan are the main livestock breeding provinces in China,so the Government should provide more financial funds for the development of the livestock industry in the central region and give full play to the role of financial funds in the high-quality development of the livestock industry in the centre.

Table 9. Heterogeneity regression results of factors influencing the high quality development of China's animal husbandry industry

Variables	eastern		western		centre		northeast	
	FE_TW	PCSE	FE_TW	PCSE	FE_TW	PCSE	FE_TW	PCSE
PGDP	0.123**	0.00498	0.156**	0.145***	-0.0810***	-0.0605**	0.117	0.0692
	(0.0508)	(0.0308)	(0.0697)	(0.0494)	(0.0257)	(0.0284)	(0.206)	(0.0705)
URB	1.126***	0.282***	0.689*	0.830***	-0.0514	0.0237	0.264	0.128

	(0.283)	(0.0989)	(0.387)	(0.304)	(0.115)	(0.0840)	(0.408)	(0.247)
TEC	-0.00818	0.0553***	0.0380***	0.0430***	0.0147	0.0145**	0.00454	0.0485**
	(0.0213)	(0.0207)	(0.0103)	(0.00964)	(0.00886)	(0.00641)	(0.0459)	(0.0238)
TRA	0.135**	-0.0186	-0.0141	-0.00770	0.0614***	0.0601***	-0.142	-0.226***
	(0.0621)	(0.0274)	(0.0158)	(0.0104)	(0.0162)	(0.0166)	(0.0956)	(0.0648)
IND	1.262*	0.329*	0.508	0.625**	0.195	0.0941	0.0295	0.0200
	(0.712)	(0.177)	(0.330)	(0.259)	(0.122)	(0.0869)	(0.122)	(0.0852)
FIN	0.00850*	0.0197***	0.0205***	0.0223***	0.000393	-2.02e-05	0.0237*	0.0156**
	(0.00507)	(0.00643)	(0.00492)	(0.00509)	(0.00270)	(0.00281)	(0.0122)	(0.00699)
ARI	-0.0797	-0.198**	0.381***	0.398***	0.194***	0.186***	-0.0643	0.0501
	(0.0907)	(0.0783)	(0.0705)	(0.0723)	(0.0305)	(0.0205)	(0.0913)	(0.0576)
POD	0.256**	0.0540***	2.486***	2.422***	0.277	0.133	0.566	1.341
	(0.126)	(0.0104)	(0.391)	(0.406)	(0.364)	(0.362)	(1.511)	(1.151)
PFA	-0.216	0.110	0.221	0.244*	0.0548	0.0376	0.435*	0.118
	(0.413)	(0.476)	(0.191)	(0.134)	(0.0668)	(0.0501)	(0.243)	(0.117)

4. Conclusions and Suggestions

4.1. Conclusions

Drawing upon panel data from the animal husbandry industry between 2010 and 2022,this paper constructs the evaluation indexes for high-quality animal husbandry development.These indicators consist of five key dimensions:output efficiency,product safety,resource conservation,environmental friendliness,and scientific and technological ＆management.Employing both entropy weight and cluster analysis methodologies,the study measures and comprehensively ranks the level of high-quality development in the livestock industry across 30 provinces in China over the period from 2010 to 2022.In addition,the research explains regional differences,characteristics of temporal dynamic evolution and spatial association network characteristics of high-quality development of China's livestock industry through the utilisation of Dagum's Gini coefficient,kernel density diagrams,Markov Chain Transfer Matrices and Social network analysis method.Finally,the study seeks to identify and appraise the macro-level factors that affect the high-quality development of animal husbandry in China,finally presenting a set of key findings.

Firstly,the level of high-quality development in China's animal husbandry industry exhibits a trend of slow growth.When categorised by dimension,this growth is reflected as follows:the level of scientific and technological ＆management surpasses that of environmental friendliness,which exceeds resource conservation.Output efficiency and,finally,product safety follow.Regionally,Shanghai and Jiangsu stand out as high-quality livestock development areas.Five provinces–Anhui,Beijing,Fujian, Yunnan,and Chongqing–comprise the medium-quality tier.Eleven provinces, specifically Gansu,Guangdong,Guangxi,Guizhou,Hainan,Henan,Heilongjiang,Hubei, Inner Mongolia,Ningxia,and Qinghai,constitute the medium-low quality group.The remaining twelve provinces,Hebei,Hunan,Jilin,Jiangxi,Liaoning,Shandong,Shanxi,Shan xi,Sichuan,Tianjin,Xinjiang,and Zhejiang,represent the low-quality tier.

Secondly,regional differences in the level of high-quality development in China's animal husbandry industry are minimal and demonstrate a slight contraction.Intra- regional differences adhere to an East > Central > West pattern,while inter-regional differences follow an East and Central > East and West > Central and West.The contribution of these

differences to the development has transitioned from an inter-region > intra-region > hypervariable density pattern in 2010 to a hypervariable density > intra-region > inter-region pattern in 2022.The effect of intra-regional differences on overall differences remains relatively stable.However, the effect of inter-regional differences on overall differences is decreasing,while the effect of hypervariable density is rising in significance.

Thirdly,from the temporal dynamic evolution trend,the level of high-quality development of the livestock sector has increased in the eastern,central and western regions..The probability of each region maintaining its current level of high-quality livestock development is high.Advancements in high-quality livestock development primarily occur between adjacent levels,with cross-stage shifts being infrequent.The level of high-quality development in neighbouring areas significantly affects local development,reflecting the adage,'One who is near vermilion is stained red,one who is near ink is stained black.'The probability of upward transition is highest when a low-level area borders a medium-low level area,when a medium-low level area neighbours a medium-level area,and when a medium-level area is adjacent to a high-level area.

Fourth,China's high-quality development of the animal husbandry industry is characterised by significant spatial correlation network accessibility and multiple overlapping phenomena.The”trickle-down effect”is greater than the”Siphon effect”in major livestock-producing provinces such as Heilongjiang,Jilin,Liaoning,Inner Mongolia, Xinjiang,Shanxi,Shaanxi,Sichuan,Gansu and Yunnan,while the”Siphon effect”is greater than the”trickle-down effect”in economically and socially developing provinces such as Beijing,Shanghai,Jiangsu and Zhejiang.

Fifthly,the level of high-quality development in the livestock industry is subject to the effect of several factors,including urbanisation rate,the level of scientific and technological innovation,the level of transport infrastructure,industrial structure,the level of agricultural financial development,agricultural industry structure,and population density.Higher levels of urbanisation,scientific and technological innovation, transport infrastructure,agricultural financial development,and population density all correlate with higher levels of high-quality development in the animal husbandry industry.Moreover,a greater agricultural share in the industrial structure and a higher proportion of livestock in agriculture are also conducive to promoting high-quality development in the livestock industry.

4.2.Suggestions

Based on the above research conclusions, this paper argues that:

(1) Empowering High-Quality Development in Animal Husbandry Through Scientific and Technological Advancement.The livestock industry is technology-driven; therefore,scientific and technological innovation is paramount for its progression towards high-quality development.Concerning livestock and poultry germplasm resources,Chinese breeds demonstrate comparatively higher resource consumption than their foreign counterparts.Therefore,the state should prioritise the enhancement of breeding capabilities and allocate increased resources towards the research and development of superior livestock and poultry breeds.Cultivating the growth of breeding enterprises,professional associations,and cooperatives,as well as other breeding-focused economic entities,is crucial.Establishing a robust breeding system for superior livestock and poultry breeds,underpinned by collaboration between industry, universities,and research institutions,will finally lead to improved feed conversion rates and enhanced livestock and poultry production efficiency.Regarding breeding models, efforts should be directed towards the development of a modern,intelligent animal husbandry system.This system should promote intelligent animal husbandry production, integrated sales and operation networks,digitalised monitoring and management,and informatised technical services.Improvements to the animal husbandry industry's data information system are necessary to strengthen real-time data and information monitoring.In addition,investment in infrastructure is required to promote the utilisation of advanced equipment and facilities,such as breeding

environment monitoring systems,physiological sign monitoring devices,automatic feeding systems, total mixed ration preparation equipment,and automated waste treatment systems. Through these measures,the level of mechanisation and automation in the animal husbandry industry will be significantly enhanced.

(2) Cultivating an Ecologically Sound Livestock Industry through Green-led Development.It is essential to strengthen the environmental awareness of business operators and cultivate green farming practices through educational activities,training courses, and information exchange programs.These activities should equip the farming community with the knowledge and skills to implement environmentally sound animal husbandry methods,including areas such as scientific feeding and management, judicious medication use,and effective waste management.In addition,environmental protection oversight of the livestock industry must be reinforced through the establishment of a comprehensive environmental protection monitoring and enforcement tsystem.This system should include penalties for business entities responsible for significant environmental damage.Continued promotion of resource- efficient manure management is crucial.Developing an integrated agricultural model that combines farming and cultivation can facilitate the conversion of organic waste into energy and fertiliser,thereby reducing reliance on fossil fuels and promoting the ecological development of animal husbandry.

(3) A coordinated approach to promoting"grain-to-fodder"agricultural reform is required.This will necessitate shifting the current dual structure of grain and cash crops to a tripartite system including grain,cash,and fodder crops.The exploration of judicious crop rotation patterns can enhance land-use efficiency.Regionally specific models,such as silage corn,alfalfa planting,oat planting,and forage rape planting,should be evaluated. In addition,strengthening and optimising the feed industry is crucial.This can be achieved by enhancing the competitiveness of feed enterprises and encouraging feed producers to adopt low-protein diets.Besides,adjusting and optimising feed formula structures and promoting alternatives to corn and soybean meal will be advantageous. The acceleration of a modern forage grass industry guarantee system is vital.This system should be underpinned by the implementation of the national grassland ecological compensation and reward policy.Increased protection,alongside scientifically and reasonably sound grassland development,will stabilise production capacity and reduce the degradation of grassy hills and slopes.Moreover,the scientific deployment of feed formulas,tailored to the growth and development stage of livestock and poultry,is essential to ensure balanced nutrition and maximise feed utilisation.

(4) Implementing differentiated policies is key to cultivating coordinated regional development in the livestock sector.Recognising that the level of high-quality development in animal husbandry remains low in Xinjiang,Inner Mongolia,and Qinghai, the State should increase technical training and support to these regions. In addition, incentives,including tax concessions and financial subsidies,should be considered to stimulate the green and sustainable development of animal husbandry in pastoral areas. Provinces such as Sichuan,Shandong,Henan,Hebei, Hunan,Hubei,and Heilongjiang,all major livestock production hubs,also exhibit lower levels of sophisticated development in animal husbandry.In response,China should optimise resource allocation in alignment with the specific resource endowments of each region.Adhering to a breeding model that combining planting and farming,while determining appropriate levels of scale,is also recommended.Crucially,the state should implement differentiated technical support policies designed to promote technological innovation and enhance animal husbandry practices.This should be tailored to the specific technical competencies and requirements of each region.

(5) Promoting a financial service system for the livestock industry to broaden the channels of financing for the livestock industry.Firstly,governmental intervention is crucial should support the development of financial services for the animal husbandry industry.Such support should include tax incentives,loan guarantees,and interest subsidies,encouraging the development of specialised financial products specifically designed

for the livestock industry.Simultaneously,these policies should aim to reduce financing thresholds and enhance accessibility for livestock breeding business entities. Secondly,a robust financial risk management system,adjusted specifically to the livestock industry,is essential.This system should incorporate rigorous risk assessment protocols and monitoring mechanisms for livestock-related projects,thereby minimising the risk exposure of participating financial institutions.Thirdly,the integration of financial technologies in livestock financial services should be encouraged to optimise efficiency and accessibility.This comprises the development of innovative financial service delivery mechanisms,including the establishment of online platforms to facilitate convenient access.Moreover,educational programmes focusing on financial literacy in the livestock industry are vital to enhance the understanding and service quality of financial practitioners operating in this sector.Finally,stringent supervisory measures and comprehensive evaluations of financial services adjusted to the livestock industry are necessary. These measures will ensure the transparency,accountability,and stability of the financial service system,finally contributing to the sustainable growth and development of the livestock industry.

Author Contributions: Conceptualization,C.L.and T.S.;methodology,C.L.;software,T.S.;validation, C.L.;formal analysis,T.S.;investigation,T.S.;resources,C.L.;data curation,T.S.;writing—original draft preparation,T.S.;writing—review and editing,C.L.;supervision,C.L.;funding acquisition,C.L.All authors have read and agreed to the published version of the manuscript.

Funding: This work was funded by the National Natural Science Foundation of China (The effect of infant milk powder safety trust index on product competitiveness—Index measurement, Correlation model construction and market simulation, Project number 71673042)

Institutional Review Board Statement: This study mainly focused on models and data analysis and did not involve human factors considered dangerous. Therefore, ethical review and approval were waived for this study.

Informed Consent Statement: Not applicable.

Data Availability Statement: The data presented in this study are available on request from the corresponding author.

Acknowledgments: We also appreciate the constructive suggestions and comments on the manuscript from the reviewer(s) and editor(s).

Conflicts of Interest: The authors declare no conflict of interest.

References

1. Wang,M.L.,Li,P.C.,& Ma,X.P.The Influence of Scale Structure Adjustment on the High-quality Development of Animal Husbandry and Its Path Optimization: An Analysis from the Perspective of Pig Breeding Scale.Chinese Rural Econ,2022,12-35.
2. Li,J.G.;Yang,W.H.;Liu,L.L.;Liu,X.M.;Qiu,F.D.;Ma,X.D.Development and environmental impacts of China's livestock and poultry breeding.J.Clean Prod.2022,371,133586.DOI:10.1016/j.jclepro.2022.133586.
3. Qian,Y.;Song,K.H.;Hu,T.;Ying,T.Y.Environmental status of livestock and poultry sectors in China under current transformation stage. Sci.Total Environ.2018,622,702-709.DOI:10.1016/j.scitotenv.2017.12.045.
4. Zhang,Y.,Huang,Y.;Liu,Y.Strategic Thinking on Developing Grassland Agriculture to Ensure China's Food Security under the New Situation. Strategic Study of CAE.2023,25,73-80.DOI:10.15302/J-SSCAE-2023.04.007.
5. Feng,D.M.;Mao,K.;Yang,Y.J.;Hu,Y.Crop-livestock integration for sustainable agriculture in china: the history of state policy goals, reform opportunities and institutional constraints.Front.Agric.Sci.Eng.2023,10,518-529.DOI:10.15302/J-FASE-2023525.
6. Peng,J.F.;Zhou,Y.L.;Zhang,Z.Y.;Luo,Y.C.;Cheng,L.The development logic,scientific Connotation,and promotion path of rural eco-industries in China.Heliyon.2023.DOI:10.1016/j.heliyon.2023.e17780.
7. Zhang,L.L.;Wang,Y.F.;Dunya,R.How Does Environmental Regulation Affect the Development of China's Pig Industry. Sustainability.2023,15,8258. DOI:10.3390/su15108258.
8. Yu,F.W.;Huang,X.;Wang,G.L.High-quality Development of Animal Husbandry:Theoretical Interpretation and Realization Path.Chinese Rural Econ.2021,4.
9. Wang,M.L.China's Livestock Industry Development:Achievements,Experiences and Future Trends.Issues Agric Econ.2018, 08,60-70.DOI:10.13246/j.cnki.iae.2018.08.007.
10. Liu,G.;Luo,Q.F.;Zhang,L.Y.The Development of Animal Husbandry Since 40 Years of Reform and Opening-up:Achievements, Challenges and Countermeasures.Chinese Rural Econ.2018,12,19-36.
11. Li,C.L.;Du,X.;Wang,G.Y.High-qulity development of animal husbandry:Framework,key points and policy design.J.China Agric.Univ.2023,28(08),296-305.DOI:10.11841/j.issn.1007-4333.2023.08.24.

12. Li,J.;Pan,L.S.Main Contradictions and Solutions to the High-Quality Development of Animal Husbandry under the Background of Rural Revitalization.Econ Review J.2022,8.DOI:10.16528 /j.cnki.22－1054 /f.202208058.
13. Wang,G.G.;Yang C.;Wang,M.L.Measurement and Spatial Distribution of the Development Level of Animal Husbandry's Modernization in China.J.Huazhong Agric.Univ(Soc.Sci.Ed).2018,6,137.DOI:10.13300/j.cnki.hnwkxb.2018.06.002.
14. Xiong,H.;Xiong,X.Z.;Wang,M.L.Measurement of China's animal husbandry modernization development level and the diagnosis of obstacle factors.J.Hunan Agric.Uni(Soc.Sci).2023,24,1.DOI: 10.13331/j.cnki.jhau(ss).2023.01.006.
15. Lu,H.G.;Zheng,J.X.;Chen,C.B.;Wei,Z.Study on the Evaluation of High Quality Development Level of Animal Husbandry in Hubei Province and its Coupling Coordination*——Based on the Perspective of Industrial Chain.Chinese J.Agric Resour Reg Plann.2022,43,5.DOI:10. 7621/cjarrp. 1005-9121. 20220525.
16. Zhang,Z H.;Ji,Y.F.;He,Z.J.Evolution in the Spatio-temporal Pattern on Animal Husbandry Green Development Competitiveness in China.J.Agric.Sci.Technol.2022,24,12-23.DOI:10.13304/j.nykjdb.2021.0432.
17. Xiong,X.Z.;Xiong,H.;Ma,X.P.;Sun,Y.M.;Yang,C.Spatial Difference and Dyanmic Evolution of High-quality Development Level of Animal Husbandry in China.Chinese J.Agric Resour Reg Plann.2022,43,113-125.DOI:10.7621/cjarrp.1005-9121.20221213.
18. Huang J,Duan X,Li Y and Guo H.Spatial-temporal evolution and driving factors of green high-quality agriculture development in China.Front. Environ. Sci. 11:1320700.doi: 10.3389/fenvs.2023.1320700
19. Wang,Y.;Kuang,Y.Evaluation,Regional Disparities and Driving Mechanisms of High-Quality Agricultural Development in China.Sustainability.2023,15,6328.https://doi.org/10.3390/su15076328.
20. Li,Q.;Liu,Z.The Spatiotemporal Impact of Digital Economy on High-Quality Agricultural Development: Evidence from China. Sustainability.2024,16,2814. https://doi.org/10.3390/su16072814.
21. Qin,S.;Han,Z.;Chen,H.;Wang,H.;Guo,C.High-Quality Development of Chinese Agriculture under Factor Misallocation.Int. J.Environ. Res.Public Health.2022,19,9804. https://doi.org/10.3390/ijerph19169804.
22. Ji,Z.H.Spatial Differences and Driving Mechanism of High Quality Development in China's Agricultural and Rural Areas.J.Quant & Tech Econ.2021,25-44.DOI:10.13653/j.cnki.jqte.2021.12.001.
23. Hao,J.H.;Gao,F.;Fang,X.Y.,Nong,X.L.;Zhang,Y.X.Multi-factor decomposition and multi-scenario prediction decoupling analysis of China's carbon emission under dual carbon goal.Sci.Total.Environ.2022,841. DOI:10.1016/j.scitotenv.2022.156788.
24. Geng,Y.Q.;Liu,L.W.;Chen,L.Y.Rural revitalization of China:A new framework,measurement and forecast.Soc.Econ.Plann Sci.2023,89.DOI:10.1016/j.seps.2023.101696.
25. Chung,Y.H.,Fare,R.,Grosskopf,S.Productivity and undesirable outputs: A directional distance function approach. J.Environ.Manage.1997,51,229-240.
26. Dagum,C.A new approach to the decomposition of the Gini income inequality ratio.Empirical Econ.1997,22,515-531. DOI:10.1007/BF01205777.
27. Zhou,F.;Wen,C.H.Research on the Level of Agricultural Green Development, Regional Disparities, and Dynamic Distribution Evolution in China from the Perspective of Sustainable Development.Agric.2023,13,1441.DOI:10.3390/agriculture13071441.
28. Shi,Z.H.;Ma,L.B.;Wang,X.;Wu,S.S.;Bai,J.;Li,Z.Y.Efficiency of agricultural modernization in China:Systematic analysis in the new framework of multidimensional security.J.Clean Prod. 2023,432,139611.DOI:10.1016/j.jclepro.2023.139611
29. Chi,M.J.;Guo,Q.Y.;Mi,L.C.;Wang,G.F.;Song,W.M.Spatial distribution of agricultural eco-efficiency and agriculture high- quality development in China.Land,2022,11,722.DOI:10.3390/land11050722.
30. Beauchemin,K.A.;Ungerfeld,E.M.;Abdalla,A.L.;Alvarez,C.Invited review: Current enteric methane mitigation options.J.Dairy. Sci.2023,105,9297-9326.
31. Lin,Z.T.;Zhang,Y.R.Temporal and Spatial Differences and Influencing Factors of Green Total Factor Productivity of Animal Husbandry in China.J.Ecol.Rural.Environ. 2023,39,1144－1157.DOI10.19741/j.issn.1673-4831.2022.09 07.
32. Xu,B.W.;Shen,Z.Y.;Lin,G.H.Evolution of green total factor productivity and regional disparity in China's husbandry.Chinese J.Eco-Agriculture,2019,27,613−622.DOI:10.13930/j.cnki.cjea.180912
33. Yang,C.;Zhu,Z.Y.;Liu,Z.Analysis on Total Factor Productivity of Regional Animal Husbandry in China.Stat & Decis.2019,35, 91-94.DOI:10.13546/j.cnki.tjyjc.2019.16.019.
34. Liu,C.M.;Fan,G.Y.;Mao,G.X.;He,P.R.Spatio-temporal variation and influencing factors of grain production efficiency in Huaihe Eco-Economic Belt in recent 20 years.J.Nat.Resour.2023, 38(3): 707-720.DOI: 10.31497/zrzyxb.20230310
35. Zhang,J.X.;Wang,H.L.Regional Differences in Carbon Emissions from Livestock Husbandry in China,Dynamic Evolution and Convergence Analysis---Based on the data of animal husbandry industry in 31 provinces (cities) from 1997 to 2017.Jianghan Trib.2020.
36. Xu,B.W.;Shen,Z.Y.;Lin,G.H. Evolution of green total factor productivity and regional disparity in China's husbandry[J]. Chinese J.Eco-Agriculture, 2019, 27(4): 613−622.DOI: 10.13930/j.cnki.cjea.180912
37. Ji,Y.F.;He,Z.J.;Li,N.J.;Li,C.;Xu,T.Green production efficiency of China's hog breeding industry: Spatial divergence and its driving factors.Plos one.2023,18,e0288176.DOI:10.1371/journal.pone.0288176.
38. Hu,C.Y.;Bo,N.;Zhao,Q.C.A study on the impact of rural finance on high-quality agricultural development in China—a test based on intermediation, threshold and spillover effects.Fron.Environ.Sci.2023, 11,1217422.DOI:10.3389/fenvs.2023.1217422.
39. Dong,J.;Li,C.B.Structure characteristics and influencing factors of China's carbon emission spatial correlation network: A study based on the dimension of urban agglomerations.Sci.Total Environ.2022,853,158613.DOI:10.1016/j.scitotenv.2022.158613.
40. Qu,J.S.;Han,J.Y.;Liu,L.;Xu.L.;Li,H.J.;Fan,Y.J.Inter-provincial correlations of agricultural GHG emissions in China based on social network analysis methods.China Agric Econ.Rev.2021,13,229-246.

41. Zhou,Z.;Duan,J.Q.;Geng,S.Q.;Li,R.Spatial Network and Driving Factors of Agricultural Green Total Factor Productivity in China.Energies.2023,16,5380.DOI:10.3390/en16145380
42. Huo,T.F.; Cao,R.J.; Xia,N.N.;Hu,X.;Cai,W.G.;Liu,B.S.Spatial correlation network structure of China's building carbon emissions and its driving factors:a social network analysis method.J.EnvironManage.2022,320,115808.DOI:10.1016/j.jenvm an.2022.115808
43. Fan,JD.;Xiao,Z.h.Analysis of spatial correlation network of China's green innovation.J.Clean.Prod.2021,299,126815.DOI: 10.10 16/j.jclepro.2021.126815
44. Cheng,L.;Huang,L.X.;Yan,Y.C.;Wen,C.H.Resarch on the Measurement and Influencing Factors of Rural Ecological Resilience in the Yangtze River Economic Belt*.Chinese J.Agric Resour Reg Plann.2023.
45. Yang,X.J.The Spatial-temporal Evolutionand Driving Factors of China ' s Common Prosperity.Shanghai J.Econ.2023,11,5-24.DOI:10.19626/j.cnki.cn31-1163/f.2023.11.001.
