# Supplementary material for: Spatial-temporal characteristics and influence factors of high-quality development of animal husbandry industry in China
Source: PLoS One. 2025 Feb 4;20(2):e0313906. doi: 10.1371/journal.pone.0313906 (PMC11793817; doi:10.1371/journal.pone.0313906)
Supplement: Attachment — Submitted filename: Response to Reviewers.docx [file pone.0313906.s003.docx]

We thank the reviewers for their comments. I feel that the quality of the article has been greatly improved after I revised it according to the reviewers' comments.

**Point1:**Introduction: The current introduction primarily focuses on the research background and existing literature, but it is somewhat weak in explaining the theoretical framework. It is recommended to add a section in the introduction specifically to elaborate on the theoretical framework of this study. This framework should address the following:
o Why were these five dimensions (output efficiency, product safety, resource conservation, environmental friendliness, and technological management level) chosen to evaluate the high-quality development of the livestock industry?
o How are these dimensions interrelated?
o How does this theoretical framework connect with existing theories of sustainable development or high-quality development? A clear theoretical framework will help readers better understand the logic and significance of the research.

**Author response1:**

The theoretical part of this paper mainly focuses on the construction of indicators in 2.2.

The reason why these five dimensions are used to construct the evaluation indicators for the level of high-quality development of the livestock industry is mainly due to the reference to the document issued by the General Office of the State Council of China in 2020 - Opinions of the General Office of the State Council on Promoting the High-Quality Development of the Animal Husbandry Industry, an authoritative document which explicitly puts forward the need to form a ‘new pattern of high-quality development that is highly efficient in terms of output, safe in terms of products, resource-saving, environmentally friendly, and effective in terms of regulation and control’. As the indicators related to ‘effective regulation’ are difficult to obtain, coupled with the fact that the level of science and technology ＆management are crucial to the high-quality development of the livestock industry, this paper selects indicators from both the level of science and technology and the level of management of the livestock industry. In turn, it constructs the evaluation index of the level of high-quality development of animal husbandry. Undeniably, due to the availability of data, the level of high-quality development of animal husbandry constructed in this paper is bound to have limitations, but in the construction of the indicators always follow the principles of scientific, comprehensive, systematic and complete construction of indicators, and try to be as close as possible to the reality of China's animal husbandry development, and therefore can also be a certain degree of response to China's high-quality level of development of the animal husbandry industry.

In the selection of specific indicators, the main reference was to existing research on the modernisation and green development of the livestock industry.

The four indicators of high output efficiency,product safety,resource conservation and environmental friendliness are parallel to each other.The science and technology ＆management levels are the internal driving forces that can promote the synergistic development of output efficiency, product safety, resource conservation and environmental friendliness. However, in this paper, this indicator of science and technology ＆management level is used as a supplementary indicator to evaluate the high-quality development level of animal husbandry together with output efficiency, product safety, resource conservation and environmental friendliness.

**Point2:**Graph and Chart Optimization: Although the graphs and charts in the article provide a wealth of information, there is room for improvement:
o Figures 1 and 2: It is recommended to use different colors or patterns to distinguish the various indicators, enhancing readability. Additionally, consider adding trend lines to more clearly display the changes over time.

**Author response:**Thanks to the reviewers for their comments. Figures 1 and 2 do have some problems and I have improved them. Figures 1 and 2 are line graphs with different colours used for each fold line. On top of that, trend lines were added and it is easy to see the trend of the different fold lines.

o Figures 4 and 5: These two figures contain a lot of information, which may confuse readers. It is suggested to split them into multiple sub-figures, with each sub-figure focusing on a specific region or comparison, to more clearly display the differences between regions.

**Author response:**Thanks to the reviewers for their comments. However, I think splitting it up would be a bit of a waste of space. In addition, putting the three lines of intra-regional differences and the three lines of inter-regional differences together would be more able to compare the intra-regional differences of East, Central and West and the inter-regional differences of East-Central, Central-West and East-West.

o Tables 2 and 3: Consider using a heatmap to display the data, which would more intuitively show the differences and trends across different provinces.

**Author response:**We thank the reviewers for their comments. I think these two tables are a clear presentation of what I want to say. For table 2, the heat map is not able to show the average annual growth rate. So here in this paper I still want to present it in the original table.

**Point3:**Targeted Policy Recommendations: The current policy recommendations are somewhat broad and general; they could be made more targeted:
o For each major finding, provide corresponding specific policy recommendations. For example, based on the finding that the "technological management level" dimension scores the highest, suggest how to further enhance and promote advanced management practices.(**From this section, it can be seen that the production efficiency and resource utilisation efficiency of China's high-quality livestock development is still very low, therefore, the policy recommendation of this paper is to prioritise the promotion of the production efficiency of the livestock industry.The level of product safety is also low, hence the policy recommendations to ensure product safety.)**
o Propose differentiated development strategies based on the characteristics of different types of regions (high-quality, medium-quality, medium-low quality, and low-quality)**.(From this part, it can be seen that the level of high-quality development of the livestock industry in China's main livestock- producing provinces is still very low; therefore, suggestions such as focusing on the main livestock-producing provinces and giving policy preferences to the main livestock-producing provinces are put forward.）**
o Based on the results of the spatial correlation network analysis, suggest how to promote regional collaborative development**(The policy recommendations for this section are to strengthen regional linkage development and to take advantage of the spatial spillover effects of developed provinces.).**
o Discuss in detail how to promote the high-quality development of the livestock industry by adjusting these factors (such as urbanization rate, technological innovation level, transportation infrastructure) based on the results of the influencing factor analysis.(**Promoting the development and application of science and technology has been highlighted in the one-location proposal. This focuses on the need to sustain urbanisation and rural financial development. Urbanisation development is conducive to the large-scale development of livestock farming, and rural financial development is conducive to providing financial support for high-quality development of the livestock sector**.)
o Consider adding a timeline or roadmap that outlines how these policy recommendations should be implemented in phases. By making these improvements, the article's theoretical foundation will be more robust, the presentation of research results will be clearer and more intuitive, and the policy recommendations will be more actionable and targeted, thereby further enhancing the academic value and practical significance of the paper.

**Author response:** The policy recommendations section of the article is indeed shallow and lacks substance. Therefore, I have revised the policy recommendations of the article. The policy recommendations are mainly in the four areas of prioritising the promotion of livestock production efficiency, guaranteeing product safety, mainly promoting the level of high-quality development of livestock in the main livestock-producing provinces, promoting the increase in the urbanisation rate and the development of rural finance.

In terms of prioritising the promotion of livestock production efficiency, the main focus has been on accelerating the layout of good breed cultivation and promoting the development of production modes in the direction of scaling up and intelligentisation. In the protection of product safety, mainly from the regular vaccination of livestock and poultry and health checks; the establishment of livestock products traceability system and other aspects of the overflow of recommendations. In terms of focusing on the development of major livestock production areas, policy suggestions were made mainly in terms of the need for major livestock-producing provinces to attract human resources, and for the central government to provide more financial pens for major livestock-producing provinces. With regard to promoting the level of urbanisation and rural financial development, recommendations were made mainly in terms of strengthening compulsory education in rural areas and formulating financial products specifically designed for the high-quality development of the livestock industry.

**Point4:**Grammar:
1. There are a few errors in the use of articles, for example:
o Original: "The livestock industry stands as a pillar of the agricultural sector."
o Suggested: "The livestock industry stands as a pillar of the agricultural sector."

**Author response:**Thanks to the reviewers for their comments. It has been revised and the full text has been read through, with a focus on the correct use of articles' articles, and corrections have been made to incorrect use

2. Some sentences are overly complex and could be split:
o Original: "Driven by increasing demand for meat, eggs, milk, and other livestock products, China's livestock industry has become a cornerstone of its agricultural sector, representing 34% of agricultural output[1]."
o Suggested: "China's livestock industry has become a cornerstone of its agricultural sector, representing 34% of agricultural output[1]. This development has been driven by increasing demand for meat, eggs, milk, and other livestock products."

**Author response**:Thanks to the reviewers for their comments. The text does contain a large number of complex sentences. The text has been read through in detail and the complex sentences have been split and modified to make them easier to understand.

3. There are some inconsistencies in verb tense, for example:
o Original: "The entropy weight method hypothesizes that indicators with higher entropy values should be assigned greater weights, and vice versa."
o Suggested: "The entropy weight method hypothesizes that indicators with higher entropy values should be assigned greater weights, and vice versa."

**Author response:**Thanks to the reviewers for their comments. Revisions have been made.

**Point5:**Citation:
1. The citation format is generally correct, but some punctuation needs adjustment, for example:
o Original: "...[23-24]"
o Suggested: "...[23, 24]"

**Author response5:**Thanks to the reviewers for the heads up. The paper has been revised to cite two literature references in the text.

2. Some citations may need to be updated or supplemented:
o When discussing the latest policies (such as the 2023 revision of the "Animal Husbandry Law of the People's Republic of China"), it is recommended to directly cite official documents.

**Author response:**The citation in the text is the official document.

3. Some important points lack citation support, for example:
o "China's livestock farming is still a decentralized operation, with a short industrial chain and low economic benefits for farmers, who are unable to fully realize the production benefits brought by the agglomeration of resources." This point needs to be supported by relevant research or reports.

**Author response:**Already modified and cited

4. When discussing the development of the livestock industry in specific provinces, consider adding some local studies to strengthen the argument.

**Author response:**Thanks to the reviewer for the reminder. This paper does cite some literature in the section on province discussion in chapter 3.1, but due to my own negligence I forgot to add the references for this section. I have added the references.For example, the phrase ‘Shanghai,an international metropolis with leading scientific research institutions and a skilled workforce,prioritises environmental protection and has enacted various environmental management measures’ is largely a reference to the literature on ‘Virginie Arantes;Can Zou;Yue CheCope with waste: A government-NGO collaborative governance approach in Shanghai[J]. Journal of environmental management, 2020, 259: 109653’. The phrase”Jiangsu Province,characterised by its diverse animal husbandry models,centres its approach on scientific and technological advancements,industry upgrades,ecological farming practices,standardised management, and brand development.In addition,the province has implemented a suite of policies promoting livestock industry expansion,including financial subsidies,tax incentives,and scientific and technological support”is largely a reference to the literature on “Qi D. Transitions to Ecological Agriculture in Nan**g, China: Farm Types, Social-Political Networks, and Rural Communities[J].”Similarly,the phrase”Ningxia and Gansu face limitations in achieving a high level of high-quality development in animal husbandry due to factors such as the relative scarcity of water resources” is largely a reference to the literature on “Bin Liu;Feilian Zhang;Xiaosheng Qin;Zhe Wu;Xiaolan Wang;Yuanyuan He.Spatiotemporal assessment of water security in China: An integrated supply-demand coupling model[J].Journal of Cleaner Production, 2021, 321: 128955”.

**Point6:**When introducing research methods (such as the entropy weight method, Dagum Gini coefficient, etc.), consider citing the original or classic application literature on these methods.

**Author response6:**Thanks to the reviewers for the heads up. I have carefully studied the classical literature related to this paper with great benefit and have cited the classical literature at appropriate places. The specific citations can be seen in the research methods section of the text.

Overall, this is a high-quality research paper with significant theoretical and practical implications for understanding and promoting the high-quality development of China's livestock industry. It is recommended for major revision and subsequent acceptance for publication.

**Reviewer #2:** This paper evaluated the spatial and temporal characteristics of the quality of China's livestock development by constructing an evaluation system for the high-quality development of China’s livestock industry. The system was constructed with five principal dimensions: output efficiency, product safety, resource conservation, environmental friendliness, and the level of scientific and technological management. Moreover, the impact of the following factors on the high-quality development of China’s animal husbandry industry was investigated: GDP per capita, urbanization rate, level of scientific and technological development, level of transportation, industrial structure, level of agricultural financial development, structure of agricultural industry, population density, and financial support for agriculture. The findings indicated that the high-quality development of China’s animal husbandry industry demonstrates a notable degree of path dependence, with regional disparities exhibiting a tendency towards convergence and development in neighboring regions exerting a mutual influence. Moreover, the urbanization rate, the level of science and technology innovation, transportation infrastructure, the level of agricultural financial development, and population density were identified as exerting a positive influence on the high-quality development of China’s animal husbandry industry.
Overall, the structure of the paper was logical and the discussion and analysis were provided at a more comprehensive level. However, a few remaining issues would benefit from further revision and improvement.

We thank the reviewers for their comments. I feel that the quality of the article has been greatly improved after I revised it according to the reviewers' comments.
**Point1:**Lines 743-771 presented a comprehensive description of the impact factor analysis model. It would appear more reasonable to reclassify them as “2.1 Research Methods.”

**Author response1:**Thanks to the reviewers for the heads up. Modifications have been made. Influence factor analysis mainly used the method of multiple linear regression. On this basis, the explanatory variables were selected with reference to existing relevant studies to construct the model.

**Point2:**The description of the regions was inconsistent. For example, the country’s 30 provinces were divided into eastern, central, and western regions in lines 301-305, whereas them were described as eastern, central, western, and north-eastern regions in lines 886-887.

**Author response2:**We thank the reviewers for their valuable comments. In order to maintain the consistency of the regional division in the whole paper, the criteria for the division of East, Central, West and Northeast in the section on impact factors are changed to East, Central and West in this paper. Liaoning in the northeastern region belongs to the east, and Jilin and Heilongjiang belong to the centre. The regional heterogeneity of the influencing factors of high-quality development of the livestock industry was re-regressed. It was found that the results did not change significantly. And the new findings were further analysed sexually. See the contents of the article for specific modifications.

**Point3:**It seems more appropriate to rename the section “3. Results” as “3. Results and Discussion.”

**Author response3:**We thank the reviewers for their comments. This section has been revised

**Point4:** A number of the statements lacked a clear and direct connection to the serial number of the chart, or were not adequately supported by data or literature.
(1) The text in lines 313-315, "The overall standard ... the period from 2010 to 2022," seemed to be related to Table 2. Similarly, the phrase "rising from 0.13 in 2010 to 0.17 in 2022" in line 316 appeared to correspond to Figure 2. The terms "level of scientific and technological management" in line 317 and "level of resource conservation" in line 319 lacked the serial numbers of the charts to which they referred. There were many similar issues that required a comprehensive review of the text and the addition of graphical serial numbers that corresponded to the textual descriptions.

**Author response4:**Thanks to the reviewers for the heads up. The sentence ‘The overall standard ..... The phrase ‘between 2010 and 2022’ does relate to Table 2, and the phrase ‘from 0.13 in 2010 to 0.17 in 2022’ does relate to Figure 3. The terms ‘level of scientific and technical management’ in row 317 and ‘level of resource protection’ in row 319 are mainly relevant to Figure 2. The text has been thoroughly reviewed and the sequence numbers of the figures corresponding to the textual descriptions have been labelled.
(2) Some arguments need more supporting data or literature, such as “has remained stable but at a lower level” (line 324), “Shanghai, an international metropolis...enacted various environmental management measures” (lines 345-347), “Fujian exhibits...breeding livestock and poultry stock nationally” (lines 363-364), “Yunnan Province has...animal husbandry” (lines 373-375).
Similarly, these descriptions, “The previous study measured... its spatial and temporal evolution trends” (lines 743-744) and “Quality of transportation infrastructure...towards high-quality development” (lines 819-820), required substantiation with evidence from the relevant literature.
In conclusion, there were additional issues similar to those mentioned above that required further supplementation and improvement.

**Author response:**The statement ‘The level of output efficiency has remained stable but at a lower level’ is mainly based on the analysis of the line graph of the level of output efficiency in figure 2, and is not derived from any particular literature. The phrase“Shanghai, an international metropolis...enacted various environmental management measures” is largely a reference to the literature on “Cope with waste: A government-NGO collaborative governance approach in Shanghai[J]. Journal of environmental management, 2020, 259: 109653”.The phrase“Yunnan Province has...animal husbandry” is largely a reference to the literature on”Traditional Ecological Customary Law for Conservation and Sustainability in Biodiversity[M]//Floristic Diversity-Biology and Conservation”.

**Point5:**Some of the formatting of the text requires further standardization.
(1) There was a discrepancy in the formatting of the literature cited on lines 114 and 786 that did not align with the formatting of all other citations in the full text. The format on lines 114 and 786 was “Xiong Xuezhen (2022) [17]” and “Zhao Lei and Cheng Fang (2019)”, respectively.

**Author response:**We thank the reviewers for their comments. This section has been revised.

(2) The format “during the observation period. utilizing 2010 as a baseline” (lines 445-446) would appear to be “during the observation period, utilizing 2010 as a baseline”.

**Author response:**We thank the reviewers for their comments. There is a real problem with this sentence. The ‘u’ in ‘utilizing’ should be capitalized.

(3) The wording of “firstly” in line 796, “moreover” in line 800, and “moreover” in line 803 appeared to be more appropriate when modified to “firstly, secondly, and thirdly.”

**Author response:**We thank the reviewers for their comments. I have made changes to this section.

**Reviewer #3:** The paper constructs evaluation indicators for high quality animal husbandry development in the Chinese scenario and evaluated developments in Animal Husbandry industry in China. Adequate quantum of data, 12- year period, was evaluated. Based on the findings, the authors put forward many suggestions for animal husbandry development stressing on technological innovation, prioritizing enhancement of breeding capabilities & development of superior breeds, improving allocation for R&D, and encouraging collaborations, mechanization & automation and ecologically sound livestock operations. Agricultural reformation, changing policies and liberal financing for AH sector are also suggested. Recommendations put forward are general in nature though lack novelty. However, this scientific analysis further validated the general concepts which is the relevance of this paper. Additionally, the study put forward influencing factors on animal husbandry developments in China and findings can address issues in the current system making way for improvement in Animal Husbandry activities of China.
 Paper is well written but needs improvement on readability. All sections including results and conclusions could be more concise limiting generalized statements and repetitions.

Thank you to the reviewers for their comments. The reviewers' comments are spot on. I have read through the entire article and have revised the complex and difficult to understand sentences present in the text to make it more readable. The policy recommendations section of the article is indeed shallow and lacks substance. Therefore, I have revised the policy recommendations of the article. The policy recommendations are mainly in the four areas of prioritising the promotion of livestock production efficiency, guaranteeing product safety, mainly promoting the level of high-quality development of livestock in the main livestock-producing provinces, promoting the increase in the urbanisation rate and the development of rural finance.

In terms of prioritising the promotion of livestock production efficiency, the main focus has been on accelerating the layout of good breed cultivation and promoting the development of production modes in the direction of scaling up and intelligentisation. In the protection of product safety, mainly from the regular vaccination of livestock and poultry and health checks; the establishment of livestock products traceability system and other aspects of the overflow of recommendations. In terms of focusing on the development of major livestock production areas, policy suggestions were made mainly in terms of the need for major livestock-producing provinces to attract human resources, and for the central government to provide more financial pens for major livestock-producing provinces. With regard to promoting the level of urbanisation and rural financial development, recommendations were made mainly in terms of strengthening compulsory education in rural areas and formulating financial products specifically designed for the high-quality development of the livestock industry.

**Reviewer #4:** This is a well written manuscript the provides a deatiled insight in China's livestock industry. I enjoyed reading it and got many useful information from it. Scientifically, it is well structured and the methods that are used are sound. With minor revisions, I believe it will be appropriate for publication in Plos one journal. Please see the attached file for my suggestions.

I thank the reviewers for their comments, and I feel that the quality of this article has improved greatly after revising it according to the reviewers' comments.

1. I've changed ‘necessary’ to ‘necessity’ in the quote.
2. I have simplified the formulation of the question in the introduction. Please see the text for details.
3. I've changed ‘we’ to ‘the animal husbandry’ .
4. I've changed ‘developme nt’ to ‘development’
5. The relevant charts have been added to the legend above
6. I have read the entire text in detail and corrected a number of spelling, grammatical, and expression errors that exist in the text.I've changed ‘central’ to ‘peak’
7. I have removed the first paragraph from the conclusion and summarised the important conclusions drawn from the article in the conclusion section. The discussion section provides policy recommendations in response to the conclusions in the article.

**Reviewer #5:** The manuscript „Spatial-Temporal Characteristics and Influence Factors of high-quality development of animal husbandry industry in China“ is a complex modelling study that intends to study somehow the development of the animal husbandry industry in China. The manuscript is somehow hard to read and must be improved in language and structure. The methodology is to a large extend unclear and how it led to the results. Furthermore, are the results and the discussion mixed, which should be for readability be separated. The displays also require a major revision. For many parameters it remains open how they were obtained or calculated. In general, presentation must be improved and the aims be clarified. Language should be improved with a professional language service. Yet, presentation of aims, methods and results does not allow a full understanding of what have been conducted by the authors.

We thank the reviewers for their comments. After revising the paper according to the comments made by the reviewers, I feel that the quality of this paper has been greatly improved.This article does have some problems with the language, which I have touched up.The data presented in the paper are basically based on the original data using STATA software.The data on the level of high-quality development of the livestock industry in each province from 2010 to 2022 were calculated based on the code of the entropy weighting method by inputting the data of 25 indicators from 30 provinces from 2010 to 2022 into STATA.The Dugum Gini coefficient was calculated based on the Dugum Gini coefficient code by inputting the data on the level of high-quality development of the animal husbandry industry in 30 provinces in China from 2010 to 2022 into the STATA software. Kernel Density Plot was calculated based on the Kernel Density Plot code by inputting the data on the level of high-quality development of animal husbandry in 30 provinces in China from 2010 to 2022 into STATA software.The Markov chain model was calculated based on the Matlab code by inputting the data on the level of high-quality development of animal husbandry in 30 provinces in China from 2010 to 2022 into the Matlab software.The spatial network model was derived by inputting data on the level of high-quality development of animal husbandry in 30 provinces in China from 2010 to 2022 into the Ucinet software.The model of factors influencing the level of high quality development of the livestock sector was also simulated using STATA software.

**Point1:**Lines 8-30: A lot a free spaces are missing after comma and full stop

**Author response1:**Thanks to the reviewers for their comments. Have read through the abstract section and added spaces after commas and full stops.
**Point2:**Line 24: remove “,´” after adage

**Author response2:**Thanks to the reviewer for the heads up. It has been revised.
**Point3:**Lines 24-25: I do not get that what you intend to say here?

**Author response3:**This is a Chinese maxim.‘It means that being close to good people can make one better, and being close to bad people can make one worse, which means that the objective environment has a great influence on people.In this paper, we would like to use this famous saying to express that: in China, the level of high-quality development of animal husbandry in a province will be affected by the level of high-quality development of animal husbandry in the surrounding provinces, neighbouring provinces with high-quality development areas of animal husbandry will promote the high-quality development of animal husbandry in the region, and neighbouring provinces with low-quality development levels of animal husbandry will pull down the level of high-quality development of animal husbandry in the region. It is also used to present the law of high-quality development of China's livestock industry revealed by Markov chain.

**Point4:**Line 29: What is GDP? Please introduce abbreviations upon first usage.

**Author response4:**GDP (Gross Domestic Product) is the final result of the productive activities of all resident units of a country or region over a certain period of time. It is the core indicator of national economic accounting and an important indicator of the economic situation and level of development of a country or region.In the humanities and social sciences, it is generally known what GDP means, so it is not described in detail.Already added in the text

**Point5:**Lines 78-79: Please provide a reference for this.

**Author response5:**Thank you to the reviewers for their comments. This sentence does cite the previous literature, but in the process of writing, due to the author's negligence, he forgot to mark the references. It has been added in the text. The specific cited literature is this one: Mingli Wang.China's Livestock Industry Development:Achievements,Experiences and Future Trends[J]. Issues in Agricultural Economy.2018, 08,60-70.DOI:10.13246/j.cnki.iae.2018.08.007.

**Point6:**Lines 79-80: Please provide a reference for this.

**Author response6:**The”Since the reform and opening up,China's animal husbandry industry has made great achievements.Productivity has risen dramatically,with per capita meat,egg and milk consumption already at 140.2%,213.2% and 28.0% of the world average, respectively”quote is all from the same document which is this one:Mingli Wang.China's Livestock Industry Development:Achievements, Experiences and Future Trends[J].Issues in Agricultural Economy.2018, 08,60-70.DOI:10.13246/j.cnki. iae.2018.08.007.

**Point7:**Lines 80-82: Please provide a reference for this.

**Author response7:**This quote cites this literature:Gang Liu;QianFeng Luo;LiYang Zhang.The Development of Animal Husbandry Since 40 Years of Reform and Opening-up:Achievements, Challenges and Countermeasures[J].Chinese Rural Economy.2018,12,19-36.Additions have been made

**Point8:**Lines 82-83: Please provide a reference for this.

**Author response8:**This quote cites this literature:Gang Liu;QianFeng Luo;LiYang Zhang.The Development of Animal Husbandry Since 40 Years of Reform and Opening-up:Achievements, Challenges and Countermeasures[J].Chinese Rural Economy.2018,12,19-36.Additions have been made.

**Point9:**Lines 91-97: I do not get the sentence completely, it is too long and to detailed. Please clarify. Further, what is intended to mean “background colour” in line 92.

**Author response9:**It has been modified to the following form:The high quality of the production process is to take green development as the core, science and technology as the driving force, a safe and sufficient forage system and a high degree of attention to animal welfare as the breeding environment, and large-scale, standardised and industrialised as the mode of production.A paradigm of intensive livestock production that is highly productive, economically efficient, highly resource conservation, and environmentally friendly will eventually be developed.

**Point10:** Lines 103-116: What do you intent to communicate here to the reader?

**Author response10:**This part mainly clarifies what kind of indexes are constructed in the current literature to evaluate the level of green development or modernisation of China's animal husbandry industry, which provides reference for the construction of evaluation indexes for the level of high-quality development of the animal husbandry industry in this paper.

**Point11：**Lines 123-124: Please cite the mentioned paper.

**Author response11:**No references are cited in this section, which cites a policy issued by China - Opinions of the General Office of the State Council on Promoting High-Quality Development of the Animal Husbandry Industry - and a link to the URL of the policy document has been marked in the text.

**Point12:** Lines 123-130: The entire introduction is hard to follow, and here it is hard to get even an idea what the authors aim with this paper. I recommend that the authors should rewrite the entire introduction and the aims and make much clearer what their aims are.

**Author response12:**The introductory section has been modified somewhat. The objective of the paper has been stated at the end of the first paragraph. How these objectives are to be achieved has been stated in detail in the last part of the last paragraph of the introduction.The goal of the article is to address the following questions: what are the characteristics of high-quality development of China's livestock industry in time and space?What are the characteristics of regional differences?What factors influence the level of high-quality development in China's livestock industry?

**Point13:**Lines 133-206: For what are these methods intended to use? I remains very unclear, also what data input is used etc.

**Author response13:**Entropy weight method was used to calculate the level of high-quality development of the livestock sector from 2010 to 2020. The input data are the 25 sub-indicators of 30 provinces in 2010-2020 constructed in the previous section.Dugum Gini coefficient is used to measure the regional differences in the level of high-quality development of the livestock industry, and the input data are the data of the level of high-quality development of the livestock industry in 30 provinces in 2010-2020. Kernel Density Map and Markov Chain are used to reveal the spatial and temporal evolution characteristics of the high-quality development level of the livestock industry, and the input data are the data of the high-quality development level of the livestock industry in 30 provinces from 2010 to 2020. The spatial network model is used to reveal the spatial network linkage characteristics of high-quality development of the livestock industry, and the input data are the data of high-quality development level of the livestock industry in 30 provinces from 2010 to 2020. The multiple linear regression model is used to reveal the influencing factors of high-quality development of the livestock industry.

**Point14:** Lines 208-209: I suggest to remove political statements from the paper.

**Author response14:**Thanks to the reviewer for the reminder, but I think this sentence simply presents the reality of China's economic development and does not address the issue of political statements.This is also recognised in China's economic development.

**Point15:** Lines 212-213: Why these?

**Author response15:**The reason why these five dimensions to build the evaluation index of the level of high-quality development of animal husbandry, mainly with reference to the document issued by the General Office of the State Council of China in 2020 - ‘Opinions of the General Office of the State Council on Promoting the High-quality Development of Animal Husbandry’, which is an authoritative document clearly puts forward the formation of ‘a new pattern of high-quality development of high-efficiency output, product safety, resource conservation, environmental friendliness, and effective regulation’. This authoritative document clearly states the need to form a ‘new pattern of high-quality development with high output efficiency, product safety, resource conservation, environmental friendliness and effective regulation’. It is difficult to obtain the indicators related to ‘effective regulation and control’, plus the level of scientific and technological development and management level is crucial for the high-quality development of animal husbandry, therefore, this paper selects the indicators from the scientific and technological and management level of the animal husbandry industry. In turn, it constructs evaluation indicators for the level of high-quality development of animal husbandry. Undeniably, due to the availability of data, this paper constructs the level of high-quality development of animal husbandry must have limitations, but in the construction of indicators always follow the principles of scientific, comprehensive, systematic, and complete construction of indicators, and try to be as close as possible to the reality of the development of China's animal husbandry industry, so to a certain extent, it can also respond to the level of high-quality development of China's animal husbandry industry.

**Point16:**Lines 207-297: This section provides many data, but needs much more references.

**Author response16:**Thanks to the reviewers for the heads up. References have been added

**Point17:** Lines 298-306: Please provide links/reference to this data. It this data public available?

**Author response17:**Links to data sources were added to the article. The data in this article are publicly available. However, much of the data used in this article is based on calculations from benchmark data, such as carbon emissions, green total factor productivity in the livestock sector

**Point18:**Lines 312-422; 433-511; 526-562; 567-630; 631-735;742-959: The results are extremely hard to read and to understand and also which results are obtained by which method.

**Author response18:**The methodology used in this paper has been introduced in 2.1 Research Methods chapter section.

312-422 The content of the section is based on the high-quality development level of the animal husbandry industry in 30 provinces in 2010-2020 calculated by applying the entropy weight method based on the data of high-quality development level of the animal husbandry industry indexes selected in the text in the period of 2010-2020. Based on this, a series of analyses are carried out.

The content of parts 433-511 is based on the characteristics of regional differences in the level of high-quality development of China's animal husbandry industry in 2010-2020 calculated by applying the Dugum Gini coefficient.

The content of parts 526-562 is based on the data of China's high-quality development level of animal husbandry from 2010 to 2020, and the dynamic evolution characteristics of China's high-quality development level of animal husbandry are plotted using the kernel density map.

The content of parts 567-630 is based on the data on the level of high-quality development of China's animal husbandry industry from 2010 to 2020, and the development law of the level of high-quality development of China's animal husbandry industry from 2010 to 2020 is revealed by using the Markov chain model, and it is found that there is a ‘Matthew effect’ in the high-quality development of China's animal husbandry industry, It is found that the high-quality development of China's livestock industry has such development laws as ‘Matthew effect’, ‘path dependence’, and ‘the one who is close to Zhu is red and the one who is close to Mo is black’.

The content of parts 631-735 is based on the data of China's high-quality development level of animal husbandry from 2010 to 2020, and the network linkage characteristics of China's high-quality development level of animal husbandry from 2010 to 2020 revealed by using the social network model, and it is found that the linkage among provinces is not very strong in terms of the development of animal husbandry and other characteristics. The main livestock-producing provinces show a ‘trickle-down effect’ rather than a ‘rainbow effect’. Economically developed provinces such as Beijing, Shanghai and others show a greater ‘rainbow effect’ than a ‘trickle effect’.

Parts 742-959 are devoted to the use of multiple linear regression to reveal the factors influencing the level of high-quality development of the livestock sector.

**Point19:**Figure 1: y- and x-axis labels are missing. Furthermore, should the colour lines explained in the legend text.

**Author response19:**We thank the reviewers for their comments. Revisions have been made.The meanings represented by the different coloured lines have been described in the legend.

**Point20:**Figure 2: Axis labels are missing.

**Author response20:**We thank the reviewers for their comments. Revisions have been made.

**Point21:**Table 2: What are the numbers express in the table per year? How are the ranks estimated? Is the growth rate per 2year cycle calculated and averaged over the entire number of cycles or is it the annual mean when only considering 2010 and 2022?

**Author response21:**The figures for each year represent the level of high-quality development of the livestock sector calculated for each province each year. The sorting is the ranking of each province among 30 provinces each year. For example, the 2 in the third column of the third row indicates that Beijing ranked second out of 30 provinces in 2010 in terms of the level of high-quality development of the livestock industry.The average annual growth rate is based on data for 2010 and 2020 using the power formula in excel.

**Point22:**Based on which criteria were the categories set, i.e. why is 0.25-0.31 high quality and why not another higher number? Why are the categories not same sizes, i.e. ranging from 0.04 to 0.06. Why are the highest two categories referred to livestock industry and the lower two to animal husbandry? What is “medium-low” ?

**Author response22:**The four classifications were derived using Q-cluster analysis. This is shown in the figure below. Table 3 of this paper is derived by applying the summary of the following figure. The four classifications are derived by averaging the level of high-quality development of animal husbandry in 30 provinces from 2010 to 2022. There are indeed problems with ‘livestock industry’ and ‘animal husbandry industry’ in the classification column. According to this paper, ‘animal husbandry industry’ should be applied. Therefore, this paper replaces all ‘livestock industry’ with ‘animal husbandry industry’.

Both the Livestock Industry and the Animal Husbandry Industry are involved in animal husbandry and agricultural activities, but they differ in scope and focus.

Livestock industry is a broader term that encompasses all activities related to the raising of livestock, including, but not limited to, pig raising, cattle raising, sheep raising, and poultry raising. The term emphasises the production activities of the livestock industry, including rearing, breeding, and marketing of the final products, such as meat, dairy products, eggs, and so on.

Animal Husbandry Industry, on the other hand, focuses more on agricultural management practices, including the scientific rearing and management of livestock. It includes not only the raising of livestock, but also involves methods and techniques to improve livestock productivity, such as scientific feeding, disease prevention and control, etc.23 Animal Husbandry Industry, which can also be translated as agro-pastoralism, emphasises the management and utilisation of animals for maximum economic benefit .

In general, Livestock industry focuses more on livestock production activities and economic activities, while Animal Husbandry Industry focuses more on agricultural management practices and technology applications to improve livestock productivity and product quality. Both are important components of the agricultural economy, but with a slightly different focus.

The provinces in the above are marked in Chinese. These provinces are in order from left to right:jilin、xinjiang、hebei、shanxi、jiangxi、liaoning、shanxi、hunan、shandong、sichuan、zhejiang、tianjin、gansu、hainan、guangdong、ningxia、henan、guangxi、hubei、Inner Mongolia、guizhou、heilongjiang、qinghai、anhui、chongqing、fujian、yunnan、beijing、jiangsu、shanghai.

|  | 2010 | 2011 | 2012 | 2013 | 2014 | 2015 | 2016 | 2017 | 2018 | 2019 | 2020 | 2021 | 2022 | 类别 |
| --- | --- | --- | --- | --- | --- | --- | --- | --- | --- | --- | --- | --- | --- | --- |
| anhui | 0.15 | 0.14 | 0.16 | 0.18 | 0.16 | 0.19 | 0.20 | 0.21 | 0.21 | 0.23 | 0.25 | 0.25 | 0.21 | 2 |
| beijing | 0.22 | 0.21 | 0.22 | 0.23 | 0.23 | 0.24 | 0.23 | 0.22 | 0.23 | 0.23 | 0.22 | 0.28 | 0.25 | 2 |
| fujian | 0.13 | 0.14 | 0.15 | 0.16 | 0.17 | 0.19 | 0.20 | 0.21 | 0.21 | 0.22 | 0.22 | 0.22 | 0.19 | 2 |
| gansu | 0.12 | 0.13 | 0.15 | 0.15 | 0.16 | 0.17 | 0.17 | 0.19 | 0.19 | 0.20 | 0.19 | 0.20 | 0.20 | 4 |
| guangdong | 0.15 | 0.15 | 0.15 | 0.15 | 0.16 | 0.16 | 0.17 | 0.16 | 0.17 | 0.18 | 0.18 | 0.19 | 0.21 | 4 |
| guangxi | 0.13 | 0.15 | 0.15 | 0.15 | 0.16 | 0.15 | 0.16 | 0.16 | 0.15 | 0.16 | 0.18 | 0.19 | 0.19 | 4 |
| guizhou | 0.13 | 0.13 | 0.14 | 0.14 | 0.14 | 0.15 | 0.16 | 0.16 | 0.16 | 0.16 | 0.16 | 0.16 | 0.16 | 4 |
| hainan | 0.14 | 0.15 | 0.14 | 0.16 | 0.14 | 0.16 | 0.17 | 0.18 | 0.18 | 0.20 | 0.19 | 0.21 | 0.22 | 4 |
| hebei | 0.11 | 0.12 | 0.11 | 0.11 | 0.10 | 0.10 | 0.11 | 0.11 | 0.11 | 0.12 | 0.13 | 0.14 | 0.14 | 1 |
| henan | 0.09 | 0.10 | 0.10 | 0.17 | 0.18 | 0.18 | 0.19 | 0.19 | 0.18 | 0.20 | 0.22 | 0.24 | 0.16 | 4 |
| heilongjiang | 0.11 | 0.13 | 0.13 | 0.13 | 0.13 | 0.14 | 0.15 | 0.15 | 0.16 | 0.16 | 0.18 | 0.18 | 0.19 | 4 |
| hubei | 0.12 | 0.15 | 0.14 | 0.15 | 0.14 | 0.15 | 0.16 | 0.16 | 0.16 | 0.17 | 0.18 | 0.19 | 0.18 | 4 |
| hunan | 0.10 | 0.11 | 0.11 | 0.12 | 0.11 | 0.12 | 0.13 | 0.12 | 0.13 | 0.14 | 0.15 | 0.15 | 0.15 | 1 |
| jilin | 0.10 | 0.09 | 0.09 | 0.10 | 0.09 | 0.10 | 0.11 | 0.11 | 0.11 | 0.12 | 0.13 | 0.14 | 0.13 | 1 |
| jiangsu | 0.14 | 0.14 | 0.14 | 0.27 | 0.26 | 0.29 | 0.35 | 0.28 | 0.28 | 0.32 | 0.33 | 0.34 | 0.21 | 3 |
| jiangxi | 0.10 | 0.10 | 0.10 | 0.11 | 0.11 | 0.11 | 0.12 | 0.11 | 0.12 | 0.13 | 0.15 | 0.15 | 0.13 | 1 |
| liaoning | 0.10 | 0.11 | 0.11 | 0.12 | 0.13 | 0.12 | 0.13 | 0.11 | 0.12 | 0.12 | 0.13 | 0.13 | 0.14 | 1 |
| Inner Mongolia | 0.16 | 0.16 | 0.16 | 0.15 | 0.15 | 0.16 | 0.16 | 0.13 | 0.16 | 0.16 | 0.16 | 0.17 | 0.18 | 4 |
| ningxia | 0.13 | 0.14 | 0.15 | 0.14 | 0.14 | 0.16 | 0.15 | 0.16 | 0.17 | 0.18 | 0.19 | 0.20 | 0.21 | 4 |
| qinghai | 0.12 | 0.13 | 0.13 | 0.13 | 0.16 | 0.14 | 0.14 | 0.16 | 0.18 | 0.19 | 0.17 | 0.17 | 0.17 | 4 |
| shandong | 0.11 | 0.12 | 0.13 | 0.13 | 0.11 | 0.13 | 0.14 | 0.13 | 0.14 | 0.14 | 0.16 | 0.15 | 0.15 | 1 |
| shanxi | 0.07 | 0.09 | 0.09 | 0.09 | 0.08 | 0.09 | 0.08 | 0.08 | 0.10 | 0.11 | 0.12 | 0.13 | 0.13 | 1 |
| shanxi | 0.10 | 0.11 | 0.11 | 0.11 | 0.11 | 0.12 | 0.12 | 0.11 | 0.11 | 0.11 | 0.12 | 0.13 | 0.12 | 1 |
| shanghai | 0.24 | 0.28 | 0.27 | 0.36 | 0.37 | 0.35 | 0.35 | 0.37 | 0.31 | 0.31 | 0.35 | 0.29 | 0.16 | 3 |
| sichuan | 0.12 | 0.13 | 0.14 | 0.13 | 0.13 | 0.14 | 0.13 | 0.14 | 0.15 | 0.16 | 0.15 | 0.15 | 0.15 | 1 |
| tianjin | 0.15 | 0.16 | 0.13 | 0.14 | 0.13 | 0.13 | 0.13 | 0.13 | 0.13 | 0.12 | 0.13 | 0.12 | 0.12 | 1 |
| xinjiang | 0.09 | 0.10 | 0.10 | 0.10 | 0.10 | 0.11 | 0.10 | 0.12 | 0.11 | 0.12 | 0.12 | 0.13 | 0.13 | 1 |
| yunnan | 0.16 | 0.16 | 0.16 | 0.17 | 0.17 | 0.18 | 0.18 | 0.19 | 0.20 | 0.21 | 0.22 | 0.21 | 0.22 | 2 |
| zhejiang | 0.12 | 0.13 | 0.12 | 0.13 | 0.12 | 0.13 | 0.13 | 0.14 | 0.14 | 0.14 | 0.15 | 0.18 | 0.17 | 1 |
| chongqing | 0.16 | 0.17 | 0.17 | 0.18 | 0.19 | 0.19 | 0.19 | 0.20 | 0.21 | 0.23 | 0.24 | 0.23 | 0.22 | 2 |

**Point23:**Table 4: What exactly do the numbers in the table say, which dimension/scale etc and how where they obtained?

**Author response23:**Table 4 demonstrates the overall differences in the level of high-quality development of China's livestock sector in 30 provinces from 2010 to 2020, the intra-regional differences in the eastern, central and western regions, the inter-regional differences between the eastern and central regions, the central and western regions, the eastern and western regions, and the contribution of interregional variation, intraregional variation and hypervariable density to overall variation.These data were calculated based on the level of high-quality development of the livestock sector in each province from 2010 to 2020 using the Dagum Gini coefficient method, which is described in the research methodology.I think the table is clear enough.

**Point24:** Figure 3: Remove grid please. And y-axis label is missing.

**Author response24:**We thank the reviewers for their comments. Revisions have been made.

**Point25:**Figure 4: y-axis label overlaps with scale. Please remove grid.

**Author response25:**We thank the reviewers for their comments. Revisions have been made.

**Point26:**Figure 5: remove grid please and add y-axis label.

**Author response26:**We thank the reviewers for their comments. Revisions have been made.

**Point27:**Figure 6: Please add axis labels.

**Author response27:**We thank the reviewers for their comments. Revisions have been made.

**Point28:** Line 528: The usage of this software should have been mentioned already in the methods.

**Author response28:**Thanks to the reviewers for their comments, which I think are actually valuable. But in humanities and social sciences paper writing, the specifics of how the software is used are not usually mentioned.The text requires a lot of code in the course of its operation.I can provide the exact code if needed.

**Point 29:**Table 5: Please explain table better, why are categories in both, i.e. rows and columns? And with respect to the categories, see my comment to Tabl. 3

**Author response 29:**Rows 2, 3, 4, and 5 are traditional Markov chain models, i.e., Markov chain models without spatial considerations. The rest are Markov chain models under the condition of considering spatial factors. Low, medium-low,medium and high levels in the first column are neighbourhood types. For example: 0.106 in the fourth column of the sixth row represents the meaning that when the level of high quality development of animal husbandry in neighbouring provinces is low level, the probability of transferring provinces with low level of high quality development of animal husbandry to medium-low level is 0.106.All the data in this table is based on the data on the level of high-quality development of the animal husbandry industry in 30 provinces in China from 2010 to 2022 calculated using the Markov chain model.

**Point 30:**Table 7: What does numbers mean and what do numbers in brackets mean?

**Author response 30:**Thank you to the reviewers for their comments. Table 7 does have some problems with the lack of year labelling. Table 7 shows the out-degree, in-degree,closeness ,and betweenness degrees for each province for 2010 and 2020.The numbers outside of brackets in the table are sorted.For example, in the second row and second column, the number 4 (6), where 4 represents Anhui's out-degree in 2010, and (6) represents Anhui's out-degree in 2010 ranked among 30 provinces. The number 11 (5) in the sixth column of the fifth row, where 11 represents Guangdong's in-degree in 2020, and (5) represents the ranking of Guangdong's in-degree in 2020 among 30 provinces. The first four columns are for 2010 and the last four columns are for 2020. The meanings of out-degree, in-degree, closeness ,and betweenness degrees are explained in the text section above the table.Out-degree is the number of provinces exporting resources out of a province, and in-degree is the number of provinces attracting resources out of a province. The Closeness centrality can reflect the extent to which the high development of animal husbandry in a province is not controlled by other regions.The higher the value, the greater the independence.The degree of Betweenness centrality can reflect the ability of each province to control the resource elements in the network of high-quality development of the livestock industry.The higher the value, the greater the control over resources

**Point 31:**Data and abbreviations in the table are not properly explained, furthermore also indicate what stars and brackets mean, levels of significances?

**Author response 31:**Thank you to the reviewers for their comments.The abbreviations in the table are actually described in section 2.1.5.The data sources have been described in the last part of section 2.2. See the body of the article for details.

**PGDP** represents GDP per capita;**URB** represents the urbanisation rate;In addition,**TEC** denotes the level of scientific and technological development in the livestock industry,measured by the sum of agricultural patents due to the absence of separate data specifically for the livestock industry;**TRA** signifies the level of transport,measured by the transport network density in each province and calculated as the ratio of the total number of miles of railways and highways to the area of the administrative divisions of each province**;IND** represents the industrial structure, measured by the proportion of primary industry in the total sum of primary,secondary, and tertiary industries;**FIN** denotes the level of agricultural financial development, expressed by the total amount of loans for agriculture,forestry,animal husbandry,and fishery in each province;**ARI** signifies the structure of the agricultural industry, expressed as the proportion of animal husbandry output value in the total output value of agriculture;**POD** represents population density,measured by the ratio of the year-end resident population to the area of the administrative divisions in each province at the year's end.Finally,**PFA** denotes the level of fiscal support for agriculture,expressed as the percentage of local fiscal expenditure on agriculture,forestry,and water affairs in the fiscal general budget expenditures.

The meaning of the data in asterisks and brackets, and the significance levels are added below the table.

**Point 32:** Line 959: No discussion is provided. However, the results section is mixed up with discussion parts. Please separate both sections clearly, that may help to make reading of the result section better readable.

**Author response 32:**The results and discussion are grouped into one chapter, but in this chapter the results and discussion sections are split into two sub-points. I think that's pretty clear as well. There are a lot of papers presented in this format as well.The results are a summary of the key conclusions reached in the text, and the discussion is a policy recommendation in response to the main conclusions of the text.
