# Supplementary material for: Spatial-temporal characteristics and influence factors of high-quality development of animal husbandry industry in China
Source: PLoS One. 2025 Feb 4;20(2):e0313906. doi: 10.1371/journal.pone.0313906 (PMC11793817; doi:10.1371/journal.pone.0313906)
Supplement: Attachment — Submitted filename: Reviewer comments on the paper.docx [file pone.0313906.s001.docx]

**Reviewer comments on the paper, “Spatial-Temporal Characteristics and Influencing Factors of High-Quality Development of Animal Husbandry Industry in China”**

This paper evaluated the spatial and temporal characteristics of the quality of China's livestock development by constructing an evaluation system for the high-quality development of China’s livestock industry. The system was constructed with five principal dimensions: output efficiency, product safety, resource conservation, environmental friendliness, and the level of scientific and technological management. Moreover, the impact of the following factors on the high-quality development of China’s animal husbandry industry was investigated: GDP per capita, urbanization rate, level of scientific and technological development, level of transportation, industrial structure, level of agricultural financial development, structure of agricultural industry, population density, and financial support for agriculture. The findings indicated that the high-quality development of China’s animal husbandry industry demonstrates a notable degree of path dependence, with regional disparities exhibiting a tendency towards convergence and development in neighboring regions exerting a mutual influence. Moreover, the urbanization rate, the level of science and technology innovation, transportation infrastructure, the level of agricultural financial development, and population density were identified as exerting a positive influence on the high-quality development of China’s animal husbandry industry.

Overall, the structure of the paper was logical and the discussion and analysis were provided at a more comprehensive level. However, a few remaining issues would benefit from further revision and improvement.

1. Lines 743-771 presented a comprehensive description of the impact factor analysis model. It would appear more reasonable to reclassify them as “2.1 Research Methods.”

2. The description of the regions was inconsistent. For example, the country’s 30 provinces were divided into eastern, central, and western regions in lines 301-305, whereas them were described as eastern, central, western, and north-eastern regions in lines 886-887.

3. It seems more appropriate to rename the section “3. Results” as “3. Results and Discussion.”

4. A number of the statements lacked a clear and direct connection to the serial number of the chart, or were not adequately supported by data or literature.

(1) The text in lines 313-315, "The overall standard ... the period from 2010 to 2022," seemed to be related to Table 2. Similarly, the phrase "rising from 0.13 in 2010 to 0.17 in 2022" in line 316 appeared to correspond to Figure 2. The terms "level of scientific and technological management" in line 317 and "level of resource conservation" in line 319 lacked the serial numbers of the charts to which they referred. There were many similar issues that required a comprehensive review of the text and the addition of graphical serial numbers that corresponded to the textual descriptions.

(2) Some arguments need more supporting data or literature, such as “has remained stable but at a lower level” (line 324), “Shanghai, an international metropolis...enacted various environmental management measures” (lines 345-347), “Fujian exhibits...breeding livestock and poultry stock nationally” (lines 363-364), “Yunnan Province has...animal husbandry” (lines 373-375).

Similarly, these descriptions, “The previous study measured... its spatial and temporal evolution trends” (lines 743-744) and “Quality of transportation infrastructure...towards high-quality development” (lines 819-820), required substantiation with evidence from the relevant literature.

In conclusion, there were additional issues similar to those mentioned above that required further supplementation and improvement.

5. Some of the formatting of the text requires further standardization.

(1) There was a discrepancy in the formatting of the literature cited on lines 114 and 786 that did not align with the formatting of all other citations in the full text. The format on lines 114 and 786 was “Xiong Xuezhen (2022) [17]” and “Zhao Lei and Cheng Fang (2019)”, respectively.

(2) The format “during the observation period. utilizing 2010 as a baseline” (lines 445-446) would appear to be “during the observation period, utilizing 2010 as a baseline”.

(3) The wording of “firstly” in line 796, “moreover” in line 800, and “moreover” in line 803 appeared to be more appropriate when modified to “firstly, secondly, and thirdly.”
